# Chlorproguanil−Dapsone−Artesunate versus Artemether−Lumefantrine: A Randomized, Double-Blind Phase III Trial in African Children and Adolescents with Uncomplicated *Plasmodium falciparum* Malaria

**DOI:** 10.1371/journal.pone.0006682

**Published:** 2009-08-19

**Authors:** Zul Premji, Rich E. Umeh, Seth Owusu-Agyei, Fabian Esamai, Emmanuel U. Ezedinachi, Stephen Oguche, Steffen Borrmann, Akintunde Sowunmi, Stephan Duparc, Paula L. Kirby, Allan Pamba, Lynda Kellam, Robert Guiguemdé, Brian Greenwood, Stephen A. Ward, Peter A. Winstanley

**Affiliations:** 1 Muhimbili University of Health and Allied Sciences, Dar es Salaam, Tanzania; 2 College of Medicine, University of Nigeria, Enugu Campus, Enugu, Nigeria; 3 Kintampo Health Research Center, Kintampo, Ghana; 4 Department of Child Health and Paediatrics, School of Medicine, Moi University, Eldoret, Kenya; 5 Institute of Tropical Diseases Research and Prevention, University of Calabar Teaching Hospital, Calabar, Nigeria; 6 Department of Paediatrics, Faculty of Medical Sciences, University of Jos/ Jos University Teaching Hospital (JUTH), Jos, Nigeria; 7 Kenya Medical Research Institute (KEMRI)/Wellcome Trust Research Programme, Kilifi, Kenya; 8 University of Heidelberg School of Medicine, Heidelberg, Germany; 9 Malaria Research Laboratories, Institute for Advanced Medical Research and Training, College of Medicine, University of Ibadan, Ibadan, Nigeria; 10 Medicines for Malaria Venture, Geneva, Switzerland, formerly at GlaxoSmithKline, Greenford, Middlesex, United Kingdom; 11 GlaxoSmithKline, Stockley Park West, Middlesex, United Kingdom; 12 GlaxoSmithKline, Greenford, Middlesex, United Kingdom; 13 Center Muraz, Bobo-Dioulasso, Burkina Faso; 14 Department of Infectious and Tropical Diseases, London School of Hygiene and Tropical Medicine, London, United Kingdom; 15 Liverpool School of Tropical Medicine, Liverpool, United Kingdom; 16 School of Clinical Sciences, University of Liverpool, Liverpool, United Kingdom; BMSI-A*STAR, Singapore

## Abstract

**Background:**

Chlorproguanil−dapsone−artesunate (CDA) was developed as an affordable, simple, fixed-dose artemisinin-based combination therapy for use in Africa. This trial was a randomized parallel-group, double-blind, double-dummy study to compare CDA and artemether−lumefantrine (AL) efficacy in uncomplicated *Plasmodium falciparum* malaria and further define the CDA safety profile, particularly its hematological safety in glucose-6-phosphate dehydrogenase (G6PD) -deficient patients.

**Methods and Findings:**

The trial was conducted at medical centers at 11 sites in five African countries between June 2006 and August 2007. 1372 patients (≥1 to <15 years old, median age 3 years) with acute uncomplicated *P. falciparum* malaria were randomized (2∶1) to receive CDA 2/2.5/4 mg/kg once daily for three days (N = 914) or six-doses of AL over three days (N = 458). Non-inferiority of CDA versus AL for efficacy was evaluated in the Day 28 per-protocol (PP) population using parasitological cure (polymerase chain reaction [PCR]-corrected). Cure rates were 94.1% (703/747) for CDA and 97.4% (369/379) for AL (treatment difference –3.3%, 95%CI –5.6, −0.9). CDA was non-inferior to AL, but there was simultaneous superiority of AL (upper 95%CI limit <0). Adequate clinical and parasitological response at Day 28 (uncorrected for reinfection) was 79% (604/765) with CDA and 83% (315/381) with AL. In patients with a G6PD-deficient genotype (94/603 [16%] hemizygous males, 22/598 [4%] homozygous females), CDA had the propensity to cause severe and clinically concerning hemoglobin decreases: the mean hemoglobin nadir was 75 g/L (95%CI 71, 79) at Day 7 versus 97 g/L (95%CI 91, 102) for AL. There were three deaths, unrelated to study medication (two with CDA, one with AL).

**Conclusions:**

Although parasitologically effective at Day 28, the hemolytic potential of CDA in G6PD-deficient patients makes it unsuitable for use in a public health setting in Africa.

**Trial Registration:**

ClinicalTrials.gov NCT00344006

## Introduction

Malaria is clearly an insupportable burden upon sub-Saharan Africa. Approximately 210−300 million clinical malaria episodes and over 1 million deaths occur in this region annually [1]. The majority of deaths are in children under 5 years. Initiatives in vector control and avoidance have made some progress [2]. However, the efficacy of the most affordable antimalarials is compromised by the widespread emergence of *P. falciparum* resistance to these agents.

Artemisinin-based combination therapy (ACT) is clinically effective, and may reduce malaria transmission and the potential for the development and spread of resistance [3], [4]. ACT is recommended by the World Health Organization (WHO) Global Malaria Programme as first-line antimalarial treatment in Africa, though achieving an acceptable cost and dosing simplicity for ACT has been difficult [5].

Chlorproguanil−dapsone−artesunate (CDA) was developed as an affordable, fixed-dose ACT for use in Africa. Once-daily dosing over three days and good activity of the chlorproguanil–dapsone (CPG−DDS) component suggested a promising combination [6], [7]. Initial CDA studies indicated a safety profile consistent with CPG−DDS [6], [7]. Hemolysis and methemoglobinemia are known adverse effects of dapsone, and are generally mild and self limiting, though hemolysis is more pronounced in subjects with glucose-6-phosphate (G6PD) deficiency [8]. G6PD deficiency is an X-linked trait, consequently more common in men than women; heterozygous women display mosaicism [9]. Exposure to oxidative stress can induce hemolysis in G6PD-deficient subjects [10]. However, the potential for dapsone, an oxidant drug, to cause clinically relevant hemolysis in malaria patients with G6PD deficiency has not been well characterized [7], [11], [12].

Artemether−lumefantrine (AL) is the current gold standard ACT. The six-dose regimen is highly effective and represents a challenging comparator for any new agent [13]–[20]. Study objectives were to compare CDA and AL efficacy in uncomplicated *P. falciparum* malaria and further define the CDA safety profile, in particular, its hematological safety in G6PD-deficient malaria patients. A randomized, parallel-group, double-blind, double-dummy, multicenter, Phase III study was conducted to demonstrate non-inferiority of CDA to AL based on parasitological cure rate (adjusted for polymerase chain reaction [PCR] genotyping) at study Day 28. Patients were young children and adolescents, representing the most clinically vulnerable population.

## Methods

The protocol for this trial and supporting CONSORT checklist are available as supporting information; see Checklist S1 and Protocol S1.

### Ethics statement

This study was conducted in accordance with Good Clinical Practices, applicable regulatory requirements, and the Declaration of Helsinki. Approval was obtained from each participating center's ethics committee or institutional review board and the WHO Special Programme for Research and Training in Tropical Medicine. An Independent Data Monitoring Committee (IDMC) was convened and planned to conduct two interim safety analyses. The IDMC appointed an independent end-point reviewer (blinded to treatment assignment).

### Participants

Male and female patients (≥1–<15 years old) who met the following criteria were eligible for enrolment: acute uncomplicated *P. falciparum* malaria (parasite count 2000 to 200,000 µL^−1^); fever at screening or within the previous 24 h; weight ≥7.5 kg; hemoglobin ≥70 g/L or hematocrit ≥25%; willingness to comply with study procedures; written or oral witnessed informed consent from parent/guardian plus assent from patients ≥12 years old.

Subjects were excluded if they had: severe/complicated *P. falciparum* malaria; known hypersensitivity/allergy to study treatments; known G6PD deficiency, methemoglobin reductase deficiency, hemoglobin M/E, or porphyria; neonatal hyperbilirubinemia; concomitant medication associated with hemolysis or hemolytic anemia; concomitant infection (including*P. vivax*, *P. ovale* or *P. malariae*) or underlying disease that could compromise efficacy evaluation; malnutrition (weight:height ratio <−3 standard deviations or <70% of median National Center for Health Statistics/WHO normalized reference values); received treatment with another antimalarial that might have compromised study treatment evaluation, or an investigational drug within 30 days or 5 half-lives; or participated previously in the study. A negative pregnancy test was required from women of child-bearing age; breastfeeding mothers were excluded.

### Interventions

At screening, a full medical history was obtained and a clinical examination undertaken. Eligible patients were randomized (2∶1) to receive CDA (GlaxoSmithKline, Greenford, UK) 2/2.5/4 mg/kg/day once daily for three days, administered according to predefined dosing charts, or six-dose AL (Novartis Pharma AG, Basel, Switzerland). One 20/120 mg AL tablet was given for patients weighing 5–15 kg, two for 15–<25 kg, three for 25–<35 kg and four for ≥35 kg, taken at study start (Day 0), 8 h later and then twice daily for two days. AL was dosed with milk. Matching placebos were administered to maintain blinding. Administration of therapy was directly observed and if vomiting occurred within 30 mins of dosing, the patient was re-dosed. If vomiting occurred again, the patient was treated with rescue medication (as per local clinical practice guidelines) and followed until Day 42.

Patients remained hospitalized until Day 3, returning for follow-up visits on Days 7, 14, 28 and 42. Field workers visited patients at home on Days 4, 5 and 6 to ensure they remained well. Clinical evaluation and temperature recording was performed before first dose, during therapy and at all clinic follow-up visits. Subjects failing therapy at any time during the study received rescue medication immediately and were followed until Day 42 or resolution.

Venous blood samples (2 mL) were obtained for hematology assessments at screening and on Days 1, 2, 3, 7, 14, 28 and 42 and for standard clinical chemistry tests at pre-dose, on Days 3 and 42, and on Days 14 and 28 if previous assessment values were abnormal.

Asexual parasite and gametocyte counts were performed at screening, at pre-dose, every 8 h during the in-patient stay until discharge on Day 3 and at follow-up visits on Days 7, 14, 28 and 42. At each time point, two thick and one thin film were prepared and parasite densities determined by examination of a thick blood slide (10 µL thumb prick), according to WHO methods [21]. Recrudescence was distinguished from reinfection with PCR genotyping, comparing pre-dose parasites versus post-Day 7 isolates using *P. falciparum msp*-1, *msp*-2, and *glurp* [22], [23].

A pre-dose blood sample (10 µL) was collected onto pre-printed filter paper for G6PD genotype analysis at two laboratories (Shoklo Malaria Research Unit, Mae Sot, Thailand and Kenya Medical Research Institute, Nairobi, Kenya). Following DNA extraction, fragment amplification using PCR primers for loci 376 A→G, 202 G→A, 542 G→T, 680 G→T and 968 T→C allowed recognition of the wildtype (G6PD B) and the common African mutations G6PD A and A– [24]–[27]. G6PD phenotype was analyzed using pre-dose hematology samples at a central laboratory (Synexa, Cape Town, South Africa) by commercial NADPH fluorescence test (Trinity Biotech, Wicklow, Ireland). G6PD genotyping and phenotyping results were not available to investigators until after study completion.

### Objectives

The objective of this trial was to compare CDA and AL efficacy in uncomplicated *P. falciparum* malaria and further define the CDA safety profile, in particular its hematological safety in G6PD-deficient patients.

### Outcomes

The primary efficacy endpoint was parasitological cure rate (PCR-corrected) at Day 28, defined as eradication of initial malaria infection by Day 7 plus aparasitemia through to Day 28. Parasitological cure was also evaluated at Days 14 and 42 as a secondary outcome.

PCR-corrected parasitological cure was the standard method for assessing antimalarial efficacy at the time that the study was designed. However, publication of the WHO 2003 guidelines for evaluating antimalarials led to the protocol being amended to include adequate clinical and parasitological response (ACPR) as a secondary outcome [21]. ACPR and PCR-corrected ACPR (ACPRp) at Days 14, 28, and 42 were derived as per WHO 2003 definitions [21], in brief: ACPR is the absence of parasitemia, irrespective of axillary temperature without previous treatment failure. Early treatment failure is the development of severe malaria by Day 3 with parasitemia, or parasitemia on Day 2 higher than on Day 0, or parasitemia on Day 3 with fever, or Day 3 parasitemia ≥25% of Day 0 count. Late clinical failure is the development of severe malaria after Day 3 with parasitemia, or parasitemia and fever from Day 4−28, without previous early treatment failure. Late parasitological failure is parasitemia on Day 7−28 with fever, without previous early treatment failure or late clinical failure.

Other secondary outcomes included: asexual parasite and gametocyte counts; mean parasite clearance time (time at which asexual parasites became undetectable and remained so for ≥48 h); and mean fever clearance time (time at which temperature normalized and remained so for ≥48 h).

Adverse events were defined as any unfavorable and unintended sign (including an abnormal laboratory test result), symptom, or disease (new or exacerbated) temporally associated with the use of a medicinal product. Adverse events were recorded at all clinic visits and coded using MedDRA (Version 10.1). Serious adverse events were defined as: death or life-threatening illness; resulting in hospitalization or prolongation of hospitalization; significantly disabling/incapacitating; congenital anomaly; hemoglobin decrease ≥40% versus baseline; hemoglobin <50 g/L; blood transfusion; methemoglobin ≥20% or ≥10 to <20% with associated clinical symptoms; or any other event considered significant by the investigator.

#### Hemoglobin safety

A composite ‘hemoglobin safety’ endpoint was defined prospectively as: hemoglobin decrease of ≥40 g/L or ≥40% versus baseline or hemoglobin <50 g/L or blood transfusion.

### Sample size

The study was designed with ≥90% power to test the hypothesis that CDA was non-inferior to AL for the primary outcome measure. Assuming efficacy of 93% for CDA and 95% for AL, 650 and 325 evaluable patients, respectively, were required using a one-sided hypothesis test at a 2.5% significance level and a 7% non-inferiority margin. Allowing for 30% loss, target recruitment was 930∶465 patients CDA:AL. No multiplicity was associated with this single primary comparison.

### Randomization − Sequence generation

GlaxoSmithKline generated the randomization schedule comprising blocks of six patients by center.

### Randomization − Allocation concealment and implementation

Randomization was done at the study site by a telephone call to the automated Registration and Medical Ordering System (RAMOS). An individual patient's code could be accessed by an investigator via RAMOS in an emergency.

### Blinding

Investigators, technicians performing microbiological assessments and patients were blinded to study treatments.

### Statistical analysis

The intent to treat (ITT) population included all randomized patients who received at least one dose of study medication. The PP population was a sub-set of the ITT population including patients who did not violate the protocol in a way that might impact the efficacy analysis.

The primary analysis was performed on the Day 28 PP population. The difference in PCR-adjusted parasitological cure rates and the 95% confidence interval (CI) was calculated using the normal approximation to the binomial distribution. A similar analysis was performed for the ITT population and for ITT observed cases (i.e. excluding missing data). For the PP population, a logistic regression model was fitted to estimate treatment effect adjusted for country, age (<5 years, 5–<15 years), and baseline parasitemia (categorized by ≤33%, >33–<67%, and ≥67% quantile). Descriptive statistics were provided for secondary endpoints (PP and ITT populations) and safety outcomes (ITT population).

For patients who were failures owing to new infections (determined using PCR), both PP and ITT analyses deemed these subjects to be successes at that time point. However, at subsequent time points, the PP analyses considered these patients as missing and the ITT analysis assumed that they were failures.

Patients with missing data at the relevant time point were excluded from PP analyses of efficacy data (PCR-adjusted parasitological cure and ACPR). In the ITT analyses, treatment failure was imputed for missing data. For example, a patient with no Day 28 parasite count would be excluded from the primary endpoint and ACPR PP analyses but included as a treatment failure in the supporting ITT analyses. If a patient was missing a parasite assessment before Day 28, this would not affect assessment of Day 28 parasitological cure, whereas for ACPR, treatment failure could not be assessed and the patient would be excluded from the PP analysis with an imputation of failure in the corresponding ITT analysis.

Comparisons of CDA and AL for the hemoglobin safety composite endpoint and changes in hemoglobin versus baseline were analyzed by the following G6PD genotype categories: G6PD normal, G6PD heterozygous females, and G6PD deficient (hemizygous A− males and homozygous A−/A− females). Logistic regression modeling was used to quantify the impact of G6PD genotype, weight, age, baseline hemoglobin, baseline parasitemia, and treatment on hemoglobin safety endpoints.

## Results

### Participant flow

**Figure 1:**
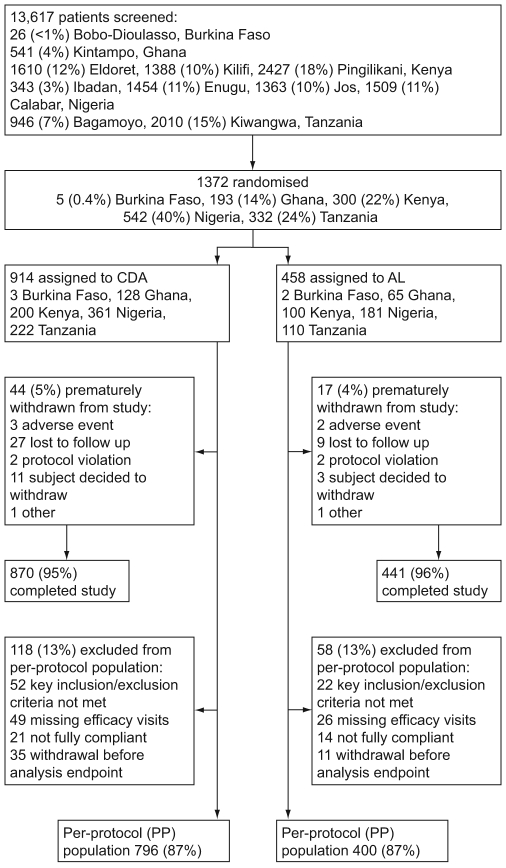
Trial profile.

Participant flow is shown in Figure 1. The ITT population comprised all 1372 randomized patients; 914 assigned to CDA and 458 to AL. The proportion of premature withdrawals was similar in each treatment group (Figure 1).

### Recruitment

The trial was conducted between June 2006 and August 2007 at health centers in Bobo-Dioulasso, Burkina Faso; Kintampo, Ghana; Eldoret, Kilifi and Pingilikani, Kenya; Ibadan, Enugu, Jos and Calabar, Nigeria; and Bagamoyo and Kiwangwa, Tanzania.

### Baseline data

Baseline characteristics were similar between treatment groups; all patients were of Black African ethnicity (Table 1). G6PD genotype was available for 1201 patients; 94/603 (16%) were A− hemizygous males and 22/598 (4%) were A−/A− homozygous females (i.e. G6PD-deficient) (Table 2). Female genotypes were significantly different from Hardy−Weinberg proportions. This is not surprising as the proportion of A− hemizygous males (in centers with data for >1 male) ranged between 2/26 (7.7%) in Ibadan and 4/19 (21.1%) in Bagamoyo. Phenotypic data were available for only 632/1372 (46%) patients.

**Table 1:** Demographic and baseline clinical characteristics of the randomized (ITT) population.

Characteristic	CDA (N = 914)	AL (N = 458)
Male sex, n (%)	457 (50)	245 (53)
Mean age, years (SD) [range]	4.2 (3.0) [1]–[14]	4.0 (2.9) [1]–[13]
Number aged 1 to <5 years, n (%)	586 (64)	300 (66)
Number aged 5 to <15 years, n (%)	328 (36)	158 (35)
Mean weight, kg (SD) [range]	16.2 (7.1) [8–62]	16.0 (6.8) [8–59]
Geometric mean parasitemia (µL^−1^) (range)	23378 (0–389415)*	22383 (185–705600)
Mean hemoglobin, g/L (SD) [range]	100 (15) [58–145]**	100 (15) [47–158]**

* Three patients in the CDA group had parasites identified at screening, were enrolled, randomized and treated, though later examination revealed that the parasites were *P. malariae* and *P. ovale*. Consequently, their *P. falciparum* parasite counts were zero.

** At some sites, screening hemoglobin level was determined using a rapid test (Haemacue, Downfield, UK), with subsequent checking using a coulter counter. Twelve subjects in the CDA group and five in the AL group had haemacue tests of ≥70 g/L with subsequent coulter counter hemoglobin values of <70 g/L. Coulter counter values are reported here.

**Table 2:** G6PD genotype (ITT population).

Category	Genotype*	CDA	AL	Total
**Males**		**(N = 457)**	**(N = 245)**	**(N = 702)**
Missing, n (%)		69 (15)	30 (12)	99 (14)
Genotype, n (%)		388 (85)	215 (88)	603 (86)
	A	85 (22)	46 (21)	131 (22)
	B	239 (62)	139 (65)	378 (63)
	A−	64 (16)	30 (14)	94 (16)
**Females**		**(N = 457)**	**(N = 213)**	**(N = 670)**
Missing, n (%)		45 (10)	27 (13)	72 (11)
Genotype, n (%)		412 (90)	186 (87)	598 (89)
	A/A	16 (4)	13 (7)	29 (5)
	A/B	101 (25)	32 (17)	133 (22)
	B/B	156 (38)	92 (49)	248 (41)
	A/A−	41 (10)	13 (7)	54 (9)
	B/A−	82 (20)	30 (16)	112 (19)
	A−/A−	16 (4)	6 (3)	22 (4)

* Percentages exclude missing data.

### Numbers analyzed

Figure 1 shows the trial profile. The PP population comprised 796/914 (87%) patients in the CDA group and 400/458 (87%) in the AL group. The primary analysis was conducted using the Day 28 PP population and 747/914 (82%) patients in the CDA group and 379/458 (83%) in the AL group were evaluable at this time point. At

Day 14, there were 787/914 (86%) evaluable patients in the CDA group and 393/458 (86%) in the AL group and at Day 42 661/914 (72%) and 332/458 (72%), respectively.

### Outcomes and estimation

#### Primary outcome: parasitological cure at Day 28

Parasitological cure (PCR-corrected) at Day 28 in the PP population was 94.1% for CDA and 97.4% for AL (treatment difference −3.3%, 95%CI −5.6, −0.9; Table 3). CDA met the non-inferiority criterion of a lower 95%CI of ≥−7%. However, an upper 95%CI of <0 indicated simultaneous superiority of AL.

**Table 3:** PCR-corrected parasitological cure at Day 28 (primary endpoint), Day 14 and Day 42 (secondary endpoints).

Parasitological cure, n (%) at:	PP population		ITT population	
	CDA (N = 796)*	AL (N = 400)*	CDA (N = 914)**	AL (N = 458)**
**Day 14**				
PCR corrected	758/787 (96.3)	376/393 (95.7)	834 (91.2)	415 (90.6)
Treatment difference (95%CI)		0.6 (−1.8, 3.0)		0.6 (−2.6, 3.9)
**Day 28**				
PCR corrected	703/747 (94.1)	369/379 (97.4)	758 (82.9)	391 (85.4)
Treatment difference (95%CI)		−3.3 (−5.6, −0.9)		−2.4 (−6.5, 1.6)
**Day 42**				
PCR corrected	591/661 (89.4)	311/332 (93.7)	647 (70.8)	336 (73.4)
Treatment difference (95%CI)		−4.3 (−7.8, −0·7)		−2.6 (−7.6, 2.4)

* In the PP analysis, patients with missing data were excluded and patients with new infections (determined by PCR) were considered successes at the first time point that this outcome was recorded, but were excluded thereafter.

At Day 14, nine patients were non-evaluable in the CDA group: two earlier new infections (0.25%), three without PCR at Day 14, and four for ‘other’ reasons. In the AL group, seven patients were non-evaluable: one earlier new infection (0.25%), five without PCR at Day 14, and one ‘other’.

At Day 28, 49 patients were non-evaluable in the CDA group: 26 earlier new infections (3.3%), ten without PCR at Day 28, and 13 ‘other’. In the AL group, 21 patients were non-evaluable: ten earlier new infections (2.5%), seven without PCR at Day 28, and four ‘other’.

At Day 42, 135 patients were non-evaluable in the CDA group: 109 earlier new infections (13.7%), ten without PCR at Day 42, and 16 ‘other’. In the AL group, 68 patients were non-evaluable: 56 earlier new infections (14.0%), five without PCR at Day 42, and seven ‘other’.

** In the ITT analysis, patients with missing data were treated as failures. Patients with new infections (determined by PCR) were considered successes at the first time point that this outcome was recorded and as failures thereafter.

Non-inferiority was maintained in the ITT analysis (Table 3). The ITT observed cases sensitivity analysis also showed CDA non-inferiority. Logistic regression performed on the Day 28 PP population suggested superiority of AL versus CDA, odds ratio (OR) 2.3 (95%CI 1.2, 4.7; *P* = 0.018), and no interaction with country, age or baseline parasitemia. The model provided overall adjusted cure rate of 95.2% for CDA and 97.9% for AL. The standard errors of these estimates were obtained using the delta method to provide the 95% confidence interval for treatment difference (−2.7%, 95%CI −4.9, −0.5). This analysis supports the primary unadjusted analysis where CDA met the non-inferiority criterion of a lower 95%CI of ≥−7%.

For parasitological secondary endpoints (Table 3), CDA was non-inferior to AL at Day 14 but not at Day 42 (PP population). Outcomes were similar in the ITT population (Table 3). PP population covariate analysis at Day 28 revealed no significant interaction with treatment for country (*P* = 0.834), age (*P* = 0.479), or baseline parasitemia (*P* = 0.179).

This study was not powered to compare treatments for individual countries. Per-country Day 28 cure rates (PP population) were as follows: Ghana/Burkina Faso, CDA 95.7% (110/115), AL 98.3% (58/59), treatment difference −2.7%, 95%CI −7.6, 2.3; Kenya, CDA 95.5% (150/157), AL 97.5% (77/79), treatment difference −1.9%, 95%CI −6.7, 2.8; Nigeria, CDA 94.1% (272/289), AL 98.0% (148/151), treatment difference −3.9%, 95%CI −7.4, −0.4); Tanzania, CDA 91.9% (171/186), AL 95.6% (86/90), treatment difference −3.6%, 95%CI −9.4, 2.2.

#### Secondary outcomes: ACPR and ACPRp

ACPR at Day 28 in the PP population was 79% for CDA and 83% for AL; ACPRp was 93% and 94%, respectively (Table 4). The proportions of early treatment failures, late clinical and late parasitological failures were similar between treatment groups (Table 4). Reinfection rates were similar for CDA and AL; 14% and 12% at Day 28, respectively, and 22% for both study drugs at Day 42 (Table 4).

**Table 4:** WHO-defined 2003 endpoints for assessing antimalarial therapy: ACPR and ACPRp.

Treatment response, n/N (%) at:	PP population		ITT population	
	CDA (N = 796)*	AL (N = 400)*	CDA (N = 914)	AL (N = 458)
**Day 28**				
Early treatment failure**	8/760 (1)	6/381 (2)	65 (7)	37 (8)
Late clinical failure	41 (5)	16 (4)	40 (4)	17 (4)
Late parasitological failure	112 (14)	44 (11)	122 (13)	49 (11)
ACPR	604/765 (79)	315/381 (83)	687 (75)	355 (78)
ACPRp†	708/765 (93)	360/381 (94)	791 (87)	403 (88)
**Day 42**				
Late clinical failure	69 (9)	28 (7)	66 (7)	28 (6)
Late parasitological failure	167 (21)	75 (19)	179 (20)	82 (18)
ACPR	527/771 (68)	275/384 (72)	604 (66)	311 (68)
ACPRp†	697/771 (90)	358/384 (93)	774 (85)	398 (87)

* For the PP population, patients with missing data or indeterminate results were excluded − the denominator shows the number of evaluable patients. For the ITT population, patients with missing or indeterminate PCR results were treated as failures. For ACPRp, in the PP and ITT analysis, patients with new infections were considered successes at that time point. However, at subsequent time points, the PP analyses considered these patients as missing and the ITT analysis assumed that they were failures.

** Early treatment failure is the same for Day 42.

† ACPRp is ACPR corrected using PCR genotyping for reinfection. Reinfection rate = ACPRp − ACPR.

#### Secondary outcomes: parasite and fever clearance

Asexual parasite counts decreased rapidly in both treatment groups; by 16 h post first treatment dose, parasite counts had declined by 99% in both groups. Mean (SD) parasite clearance time was 23.5 (11.0) h (N = 909) for CDA and 26.2 (11.5) h (N = 456) for AL (ITT population). For patients with baseline fever, mean fever clearance time (SD) was: 28.7 (23.3) h (N = 538) and 26.6 (22.2) h (N = 250) (ITT population).

#### Secondary outcomes: gametocyte prevalence

Gametocytes were present at baseline in 25/912 (3%) patients in the CDA group and in 10/458 (2%) in the AL group (ITT population). The proportion of gametoyctemic patients decreased throughout the study similarly in both treatment groups. For both the PP and ITT population analyses, the geometric means ranged between 1.0 and 1.1 parasites/µL throughout the study in both treatment groups. The median gametocyte counts were 0 prior to dosing and at each time point following dosing for both treatment groups.

### Ancillary analysis

#### Compliance with medication

A subject was considered compliant with study treatment if they received the treatment to which they were randomized as well as the correct dose for their body weight on all three dosing days. Compliance with study medication was 94% for both treatment groups.

### Adverse events

The proportion of treatment-emergent adverse events due to any cause was similar between the treatment groups: 476/914 (52%) in the CDA group and 220/458 (48%) in the AL group. The majority of adverse events were mild to moderate in intensity (96% for CDA, 98% for AL). Adverse events reported by investigators as probably or possibly drug-related occurred in 184 (20%) patients in the CDA group and 86 (19%) in the AL group (Table 5).

**Table 5:** Most frequent investigator-reported treatment-emergent drug-related adverse events reported in the ITT (safety) population (≥2% of patients in either treatment group).

Preferred term, n (%)	CDA (N = 914)	AL (N = 458)
Reticulocyte count decreased	79 (9)	44 (10)
Anemia	24 (3)	9 (2)
Hemoglobin decreased	24 (3)	7 (2)
Vomiting	14 (2)	4 (<1)
Thrombocytopenia	15 (2)	3 (<1)
Patients with at least one drug-related adverse event	184 (20)	86 (19)

Serious adverse events were reported for 63/914 (7%) patients in the CDA and 15/458 (3%) patients in the AL group. In the CDA group, serious adverse events most likely related to oxidative hemolysis occurred in 46/914 (5%) of patients versus 3/458 (<1%) in the AL group. In G6PD-deficient patients receiving CDA, 26/80 (33%) had such events (six cases of hemoglobin decreased, seven hematuria/hemoglobinuria [these were not always differentiated], two hemolysis, two hemolytic anemia and one blood methemoglobin) compared with 10/597 (2%) for G6PD normal (seven anemia, two hemoglobin decreased, one intravascular hemolysis) and 5/123 (4%) for female heterozygotes (two anemia, three hemoglobin decreased). In the AL group, malaria was the most common serious adverse event (7/458 [2%]). All serious adverse events in the AL group were in G6PD-normal patients, except one case of malaria in a heterozygous female.

#### Hemoglobin laboratory analysis

**Figure 2:**
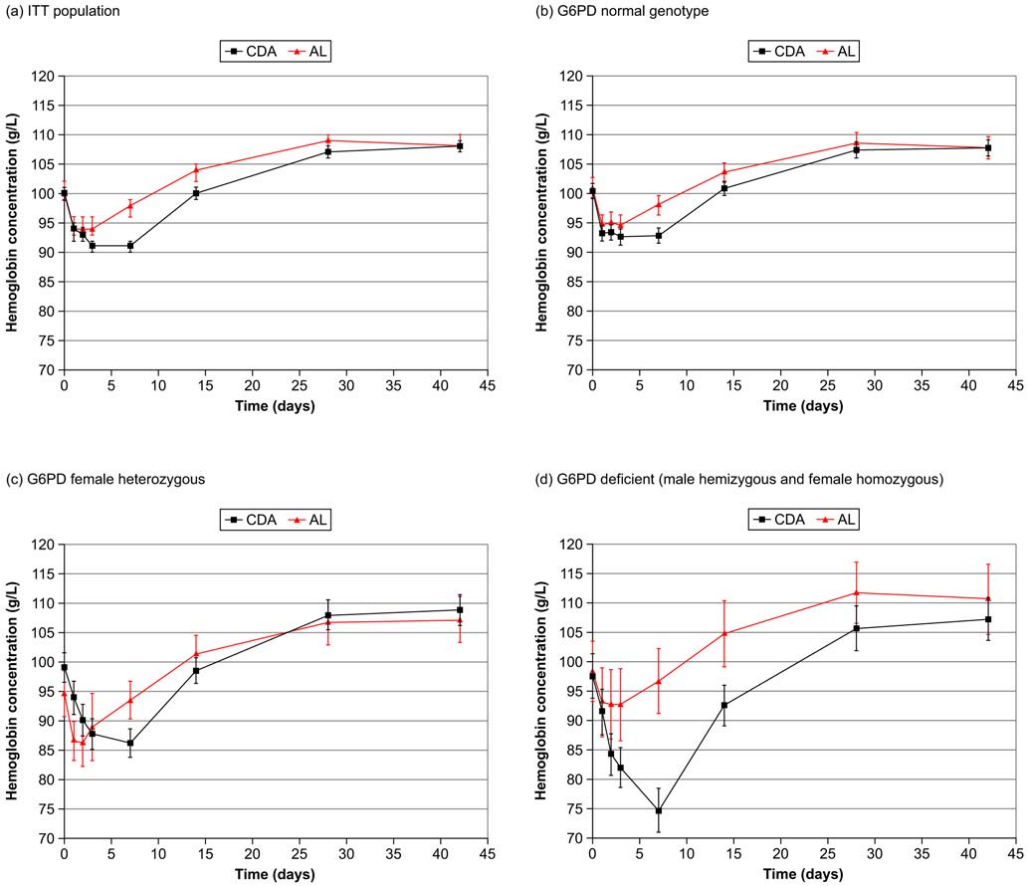
Mean hemoglobin (±95%CI) over time (a) for the ITT population and by G6PD genotype: (b) normal; (c) female heterozygous; (d) deficient (male hemizygous and female homozygous). Day 0 are screening (baseline) values.

Overall in the ITT population, mean hemoglobin concentrations decreased versus baseline from Day 1 for both treatment groups (Figure 2a), and were slightly lower for CDA versus AL on Days 3, 7 and 14 (Figure 2a). For G6PD-normal and female heterozygote patients, the maximum hemoglobin decrease was similar for both study drugs, though recovery to baseline levels was slower with CDA (Figure 2b,c). For G6PD-deficient patients, declines in hemoglobin concentration were more marked with CDA versus AL at Days 3, 7 and 14 (Figure 2d). The mean hemoglobin nadir for CDA in G6PD-deficient patients was 75 g/L (95%CI 71, 79) at Day 7 versus 97 g/L (95%CI 91, 102) for AL. The mean hemoglobin nadir in G6PD-deficient patients for AL was 93 g/L (95%CI 87, 99) occurring at Day 3 versus 82 g/L (95%CI 79, 85) for CDA.

**Figure 3:**
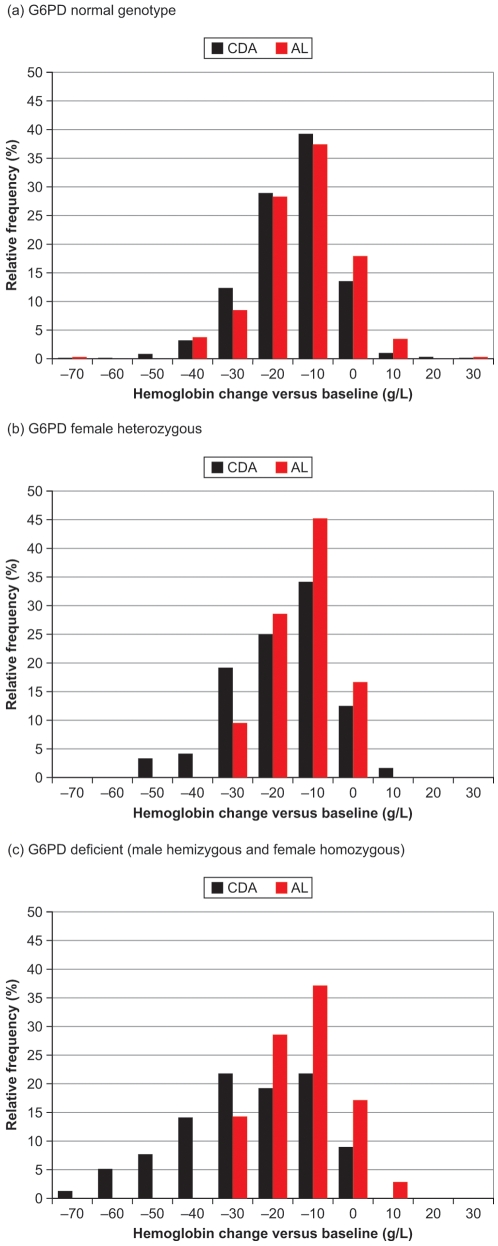
Relative frequency distribution for the maximum decrease in hemoglobin versus baseline at any time during the study, or minimum increase in hemoglobin versus baseline if hemoglobin values were greater than baseline throughout the study, by G6PD genotype: (a) normal; (b) female heterozygous; (c) deficient (male hemizygous and female homozygous).

The proportion of patients with a decrease in hemoglobin concentration versus baseline of ≥20 g/L was 305/914 (33%) with CDA and 112/458 (24%) with AL. By G6PD genotype, this endpoint occurred in 173/597 (29%) normal, 45/123 (37%) heterozygote and 45/80 (56%) deficient patients in the CDA group and in 77/322 (24%), 9/43 (21%) and 12/36 (33%), respectively in the AL group. Figure 3 shows the relative frequency distribution for maximum hemoglobin decrease versus baseline. CDA and AL had similar profiles in G6PD-normal patients (Figure 3a), whereas for G6PD-deficient patients, CDA data were left-shifted versus AL, i.e. there was a greater relative frequency of clinically significant hemoglobin decreases with CDA (Figure 3c).

#### Hemoglobin composite safety endpoint

Occurrences of the hemoglobin safety endpoint in G6PD-deficient patients were 35% (28/80) with CDA versus 0% with AL (Table 6). A post-hoc analysis for risk factors associated with occurrences of the composite endpoint found significant effects for G6PD-deficiency (OR 16.3, 95%CI 8.6, 31.2), treatment with CDA versus AL (OR 5.1, 95%CI 2.1, 12.4) and baseline parasitemia (OR 2.3, 95%CI 1.3, 4.3). There was no increased risk in G6PD heterozygous females, and no effect of age, weight or baseline hemoglobin.

**Table 6:** Occurrences of the hemoglobin safety composite endpoint (hemoglobin decrease of ≥40 g/L or ≥40% versus baseline or hemoglobin <50 g/L or blood transfusion) by G6PD genotype in the ITT (safety) population.

G6PD genotype, n/N (%)	CDA	AL
Normal	18/597 (3)	5/322 (2)
Female heterozygous	7/123 (6)	1/43 (2)
G6PD deficient*	28/80 (35)	0/36
Missing	11/114 (10)	3/57 (5)
**Total**	64/914 (7)	9/458 (2)

* Deficient is male hemizygous G6PD A− and female homozygous G6PD A–/A−.

Fourteen blood transfusions were performed for a significant decrease in hemoglobin, all in the CDA group: 12 in G6PD-deficient patients, one in a heterozygous female, and one in a G6PD-normal patient. One subject in the CDA group received a transfusion for treatment of sickle cell crisis (G6PD status unknown). A further subject in the CDA group (hemizygous male) was hospitalized on Day 6 requiring a blood transfusion (hemoglobin 38 g/L), though blood was not available. This patient subsequently recovered under close observation and supportive treatment with hematinics.

#### Mortality and withdrawals

Three deaths occurred during the study: two in the CDA and one in the AL group; none were attributed to study treatment by the investigator. In the CDA group, a 2-year-old female had severe malaria and severe herbal intoxication at Day 23 and 24 and died on Day 24; a 4-year-old male with sickle cell anemia with crisis and severe sepsis was inadvertently recruited into the study, withdrawn on Day 1 and transfused and died on Day 2. In the AL group, a 1-year-old male had severe pyrexia at Day 26 and died on Day 29. There were three other withdrawals because of adverse events, two with CDA (vomiting and febrile convulsion) and one with AL (hematuria), all of which resolved.

## Discussion

For the primary endpoint of this trial, parasitological cure at Day 28 (PP population), CDA was highly efficacious (94%). CDA was non-inferior to AL, but AL was simultaneously superior to CDA. An adjusted logistic regression analysis (PP population) also showed AL superiority. Non-inferiority of CDA to AL for the primary efficacy endpoint was not maintained at Day 42. These results confirm the high efficacy of AL in Africa [13]–[20].

It was notable that there was no difference in reinfection rates between the two treatment groups. A recent comparison of CDA and AL also found no difference in reinfection rates [28]. Lumefantrine has a half-life of 3−4 days, with concentrations above inhibitory concentrations for 10·5 days [29]. Despite the short half-life for CPG (35 h) and DDS (27 h), the duration of parasitocidal activity against susceptible parasites is approximately 3−10 days, depending on parasite strain [30]. Thus, the drugs are potentially pharmacodynamically more similar than their pharmacokinetics would suggest.

Although this study was designed primarily to assess efficacy, the most clinically important findings relate to safety, specifically G6PD-related hemolysis in the CDA group. The 2∶1 CDA:AL randomization schedule was chosen to provide adequate data for a meaningful analysis of CDA safety. Also, we defined a composite hemoglobin safety endpoint based on laboratory data for hemoglobin declines of clinical concern and blood transfusion events. Overall, the main risk factors associated with the composite endpoint were G6PD deficiency versus G6PD normal (OR 16.3, 95%CI 8.6, 31.2) and CDA treatment versus AL (OR 5.1, 95%CI 2.1, 12.4). In G6PD-deficient patients (hemizygous males and homozygous females), 35% met the composite endpoint criteria with CDA versus none with AL. Of particular concern was that 12/15 patients requiring blood transfusion were G6PD-deficient, and all in the CDA group.

This study was not powered to detect a difference in hematological safety in G6PD-deficient patients between treatment groups. However, subjects meeting the composite hemoglobin safety endpoint would clearly require hospital-based interventions. These are often difficult to access for the majority of malaria patients in sub-Saharan Africa. Based on the composite endpoint data, CDA has an unacceptable risk:benefit profile versus AL for the treatment of malaria in G6PD-deficient patients. Consequently, this population should not be exposed to CDA.

The prevalence of G6PD deficiency in this study was 16% hemizygous males and 4% homozygous females; a substantial sub-population. The lowest prevalence of G6PD deficiency was 7.7% in Ibadan, though this is considerably lower than previous estimates, generally in excess of 20% [31]. Additionally, 14% of patients were heterozygous females, only identifiable by genotyping.

G6PD phenotyping and genotyping are not generally available in Africa. Even if G6PD-deficient patients could be excluded, post-hoc regression analysis found that in G6PD-normal patients, the risk of a ≥20 g/L hemoglobin decrease versus baseline was slightly increased with CDA versus AL (OR 1.4, 95%CI 1.01, 1.96). The clinical relevance of this small difference is debatable, but further undermines CDA utility, given the availability of alternative agents. CDA was developed for use in Africa as a safe, affordable, effective and simple antimalarial. As these conditions cannot be met, the Joint Development Team decided to terminate CDA development and withdraw CPG−DDS licenses.

### Overall evidence

Although we knew that there would be some hemolytic effect of dapsone, the magnitude of the difference in this effect between CDA and AL in G6PD-deficient patients was unexpected [11]. Phase II studies with CDA in children of unknown G6PD status had been encouraging, with decreases in hemoglobin until Day 3 but with recovery by Day 7, surpassing baseline values by Day 14 [6]. There was also marginally better hemoglobin recovery in the 4 mg/kg artesunate-containing arm versus CPG−DDS alone, suggesting that the majority of the hemolysis was due to sub-optimal antimalarial therapy [6].

In this study, comparison of the timecourse for hemoglobin decline with CDA and AL is instructive (Figure 2). For AL, regardless of G6PD status, the nadir in hemoglobin concentration occurred at or before Day 3. With CDA, for G6PD normal and female heterozygotes, hemoglobin was significantly lower than with AL at Day 7 only. This was probably caused by dapsone-related shortening of erythrocyte lifespan. However, in G6PD-deficient patients, the decrease in hemoglobin with CDA was more marked, with a nadir at Day 7, and was significantly different from AL from Day 3 until Day 30. This clearly shows the effect of G6PD deficiency when oxidative and potentially hemolytic drugs are administered.

Dapsone had been used in malaria and leprosy for many years with no major safety concerns [32]–[34]. Thus, previous studies of CPG−DDS without artesunate did not prospectively include G6PD genotyping or phenotyping. Also, concerns on frequent blood sampling in young children led to less comprehensive hemoglobin assessments than in the current study. Comparison of our data with CPG−DDS clinical trials is, therefore, problematic.

In the CPG−DDS safety study by Alloueche *et al*., Day 3 and Day 14 data were sparse, but at Day 7 there was a significantly larger decrease in mean hemoglobin with CPG−DDS than for the comparator sulfadoxine−pyrimethamine (SP): treatment difference −4 g/L (95%CI −6, −2) [7]. In the current trial, the difference between CDA and AL for Day 7 mean hemoglobin was not greatly dissimilar when considering the overlap in 95%CIs: −7 g/L (95%CI −9, −4). However, the effect of the sparse hemoglobin sampling in the Alloueche *et al*. study becomes evident when the proportion of patients with hemoglobin decreases of ≥20 g/L at any time point is examined; this was 16% for both CPG−DDS and SP [7]. We know that both CPG−DDS and SP cause hemolysis, and yet the proportion of patients in the current study with hemoglobin decreases of ≥20 g/L was 33% for CDA and 24% for AL. If the data sets were comparable, we would expect CPG−DDS and SP to have results approximating those for CDA, or at least a greater proportion of clinically important hemoglobin decreases than with AL.

The position becomes even more complicated when the limited analysis of hemoglobin declines by G6PD genotype in the Alloueche *et al*. study is considered. G6PD status was assessed retrospectively and only in a subset of patients, i.e. some patients with a ≥20 g/L hemoglobin drop and matched controls (by age and sex). There was no excess risk for a ≥20 g/L hemoglobin drop with CPG−DDS versus SP in G6PD-normal or G6PD-deficient patients (classified as A− or A−/A−). However, for female heterozygotes, there was an excess risk with CD versus SP [7]. Although this analysis can provide an idea of the risk of haemolysis for CD relative to SP, it cannot give a true odds ratio for the risk of haemolysis for G6PD-deficient relative to G6PD-normal patients. Moreover, the Alloueche *et al*. results are in direct contrast to all of the G6PD-based analyses reported here, where G6PD-deficient patients were at greater risk of hemolytic events versus G6PD-normal and female heterozygote patients. Our study indicates that frequent hemoglobin assessment plus comprehensive G6PD genotype data are required for an adequate analysis of hemolytic potential in the G6PD-deficient sub-population.

A recently published study conducted in Rwanda comparing CDA versus amodiaquine (AQ) plus SP for the treatment of uncomplicated malaria is also informative [35], [36]. In this study, G6PD genotyping was available for the majority of patients. Packed cell volume (hematocrit) was evaluated, so direct comparison to our results is not possible. Although both CDA and AQ+SP caused mild hemolysis in G6PD-normal patients, hemolysis was more profound in G6PD-deficient patients with CDA, but not AQ+SP. However, in light of its continued widespread use, the risk:benefit of SP with regard to hematological safety in G6PD-normal and -deficient malaria patients needs to be properly investigated versus a non-hemolytic comparator, such as AL.

One important question is whether the hemolysis observed here is new to CDA or would have been seen with CPG−DDS versus AL. Preliminary results from a second Phase III clinical trial of similar design indicate no major differences in hematological safety between CDA and CPG−DDS (personal communication P. Winstanley).

There were some limitations in the hematological data collected in this study. A validated methemoglobin test, suitable for clinic use, was only available towards the end of the study. Consequently, only 32 patients from two centers (Eldoret and Calabar) had screening and post-baseline methemoglobin data. Collection of reticulocyte data was not automated and there was no quality control process. On examination, reticulocyte percent and absolute data could not be interpreted and the results are not presented. G6PD phenotypic data were only available for 632/1372 (46%) patients because of logistical problems in transporting samples to a central laboratory within a narrow time window and data are not presented. The difficulties in obtaining sufficient G6PD phenotype data quickly hampered assessment of safety within the G6PD-deficient population by the IDMC during the recruitment phase. For the limited data available, concordance of a G6PD-deficient phenotype with genotype in hemizygous males was 271/313 (87%), and phenotype findings supported genotype findings with respect to both G6PD-deficiency prevalence and hemoglobin safety.

### Conclusion

To our knowledge, this is the first randomized controlled clinical Phase III multi-centre study to include a component investigating the relationship between hematological safety and G6PD deficiency. There is no accepted in vitro test or in vivo model to determine the potential for G6PD-related hemolysis. G6PD genotyping and separate analysis of hemizygous males/homozygous females versus heterozygote females and G6PD-normal patients was necessary to show the clinical importance of the interaction between CDA and G6PD deficiency for hematological safety. Our results highlight that new antimalarials, such as AL, may have significant safety as well as efficacy benefits over older therapies, illustrating the need to conduct comprehensive Phase III trials of antimalarial combination therapies against relevant comparators, even when the components are already approved individually. It also highlights the challenges of developing therapies for populations harboring G6PD deficiency and possibly other polymorphisms.

## Supporting Information

Checklist S1 CONSORT checklist
(0.07 MB DOC)

Protocol S1 Trial Protocol
(0.44 MB PDF)
