# Supplementary material for: Chlorproguanil−Dapsone−Artesunate versus Artemether−Lumefantrine: A Randomized, Double-Blind Phase III Trial in African Children and Adolescents with Uncomplicated Plasmodium falciparum Malaria
Source: PLoS One. 2009 Aug 19;4(8):e6682. doi: 10.1371/journal.pone.0006682 (PMC2724683; doi:10.1371/journal.pone.0006682)
Supplement: Protocol S1 — Trial Protocol (0.44 MB PDF) [file pone.0006682.s002.pdf]

Division: Worldwide Development
Retention Category: GRS019
Information Type: Protocol Amendment

**Title:**	A multi-centre, randomised, double-blind study to compare the efficacy and safety of chlorproguanil-dapsone-artesunate versus artemether-lumefantrine in the treatment of acute uncomplicated Plasmodium falciparum malaria in children and adolescents in Africa

Compound Number: SB-714703

Effective Date: 14-JUN-2007

Protocol Amendment Number: 04

Description:

Protocol amendment to:

- Removal of tertiary objective and endpoint respectively
  - objective and endpoint considered not essential to support primary and secondary endpoints
- Correct the definitions of LPF and ACPR to be in accordance with WHO Guidelines for the Treatment of Malaria
- Changes Data Analysis and Statistical Considerations:
  - Add a detailed discussion of the choice of non-inferiority margin in line with EMEA Guideline (EMEA/CPMP/EWP/2158/99)
  - Change the analysis population for the powered key secondary analysis (superiority of CDA vs Coartem in % of patients with parasites at 24 hours) from Per Protocol to ITT in line with EMEA Guideline (EMEA/CPMP/EWP/482/99) and ICH Harmonised Tripartite Guideline (ICH E9)
  - Update Missing Data section to reflect ITT and Per Protocol principles
  - Amend proposed adverse event reporting to exclude events which are PCR-confirmed recrudescences from most planned outputs
  - Minor changes to text for purposes of clarity
- Addition of new references to relevant to the provision of the WHO reference for Adequate Clinical and Parasitological Response and choice of non-inferiority margin
- Correct the Country Specific Requirements

Copyright 2007 the GlaxoSmithKline group of companies. All rights reserved. Unauthorised copying or use of this information is prohibited.

Subject: Malaria, chlorproguanil,dapsone,artesunate

Author: Goh,Li Ean, ID MDC; Lynda Kellam; ID MDC

Revision Chronology:

GM2005/00186/00	2005-AUG-15	Original
GM2005/00186/01	2005-AUG-15	Amendment No.01: Ghana only, to add investigator and collaborator contact details as requested by their IEC.
GM2005/00186/02	2006-JAN-12	Amendment No.02: To change age of inclusion, include urine S/P testing on all subjects, and clarify provision of contraception
GM2005/00186/04	2007-APR-12	Amendment No.03:<br>• To withdraw the urine lignin test at screening and exclude any patients who have received any unknown antimalarial drugs within the past 28 days<br>• Change in protocol author and personnel responsible for study coordination<br>• Changes to Section 6.3.4 -Adequate Clinical and Parasitological Response.
GM2005/00186/05	2007-JUNE-14	Amendment No.04<br>• Change to sections 2.3 tertiary objective and 3.3 tertiary endpoint<br>• Correct the definitions of LPF and ACPR in Section 6.3.4 to be in accordance with WHO Guidelines for the Treatment of Malaria<br>• Changes to section 11 for purposes of analysis and clarity<br>• Update to section 13 to include new references relevant to the choice of the non-inferiority margin. Provision of WHO reference relevant to update in section 6<br>• Change to correct country specific requirements in Appendix 2

**Sponsor Signatory:**	**Signature:**	**Date:**
Didier Lapierre<br>Vice President, Clinical EU, HIV/AI	______________________	__________

SPONSOR INFORMATION PAGE

Clinical Study Identifier: SB-714703/005

GlaxoSmithKline R&D Ltd
Greenford Road
Greenford, Middlesex, UB6 0HE, UK
Telephone: +44 (0)20 8 422 3434

Sponsor Contact Information:

Study Physician: Dr Allan Pamba
Study Leaders: Ammar Qureshi; Dr. Cletus Ugwuegbulam

Sponsor Serious Adverse Events (SAE) Contact Information:

Case Management Group - Greenford, UK

Fax: +44 (0)20 8423 9534
E-mail Lotus Notes: UK GSK-RD-GCSP-CTSM-Admin/PharmRD/GSK
External: OAX37649@gsk.com

Regulatory Agency Identifying Number(s): Not applicable

Country Medical Departments:
East African countries:
Dr William Mwatu
GlaxoSmithKline Limited
Industrial Area, Likoni Road
P.O. Box 78392-00507 Viwandani
Nairobi, Kenya
Tel: + 254 20 534 241 or + 254 20 693 3327

West and Central African countries:
Dr Yves Aphing-Kouassi
GlaxoSmithKline Ltd
Km 16 Ikorodu Road
Ojota, Lagos
Nigeria
Tel: + 234 1 497 8152 or mobile number +234 802 518 5479

INVESTIGATOR AGREEMENT PAGE

I confirm agreement to conduct the study in compliance with the protocol, as amended by this protocol amendment.

Investigator Name: ______________________________

______________________________ ______________________________
Investigator Signature Date

Co-investigator Name:
____

______________________________ ______________________________
Investigator Signature Date

TABLE OF CONTENTS

PAGE

ABBREVIATIONS ... 9

PROTOCOL SUMMARY ... 11

1. INTRODUCTION ... 13
 1.1. Background ... 13
 1.2. Rationale ... 14

2. OBJECTIVE(S) ... 15
 2.1. Primary ... 15
 2.2. Secondary ... 15

3. ENDPOINT(S) ... 15
 3.1. Primary ... 15
 3.2. Secondary ... 16

4. STUDY DESIGN ... 16

5. STUDY POPULATION ... 17
 5.1. Number of Subjects ... 17
 5.2. Eligibility Criteria ... 17
  5.2.1. Inclusion Criteria ... 17
  5.2.2. Exclusion Criteria ... 18
  5.2.3. Other Eligibility Criteria Considerations ... 19

6. STUDY ASSESSMENTS AND PROCEDURES ... 19
 6.1. Demographic and Baseline Assessments ... 19
 6.2. Safety ... 20
  6.2.1. Clinical assessment and vital signs ... 20
  6.2.2. Haematology and clinical chemistry ... 20
  6.2.3. G6PD analysis ... 20
  6.2.4. Pregnancy ... 21
   6.2.4.1. Time period for collecting pregnancy information ... 21
   6.2.4.2. Action to be taken if pregnancy occurs ... 21
  6.2.5. Home visits ... 22
 6.3. Efficacy ... 22
  6.3.1. Parasitology ... 22
  6.3.2. Parasite DNA analysis ... 23
  6.3.3. Parasitological Cure Rate ... 23
  6.3.4. Adequate Clinical and Parasitological Response ... 23
  6.3.5. Clearance times (PCT and FCT) ... 24
 6.4. Urine testing for antimalarial drugs ... 24
 6.5. Pharmacokinetics ... 25

7. INVESTIGATIONAL PRODUCT(S) ... 25
 7.1. Description of Investigational Product ... 25

7.2. Dosage and Administration 26
7.3. Dose Rationale 26
7.4. Blinding 26
7.5. Treatment Assignment 27
7.6. Packaging and Labelling 27
7.7. Preparation 28
7.8. Handling and Storage 28
7.9. Product Accountability 28
7.10. Assessment of Compliance 28
7.11. Treatment of Investigational Product Overdose 29
7.12. Occupational Safety 29

8. CONCOMITANT MEDICATIONS AND NON-DRUG THERAPIES 30
8.1. Permitted Medications 30
8.2. Prohibited Medications 30
8.3. Non-Drug Therapies 30

9. SUBJECT COMPLETION AND WITHDRAWAL 30
9.1. Subject Completion 30
9.2. Subject Withdrawal 31
9.2.1. Subject Withdrawal from Study 31
9.2.2. Subject Withdrawal from Investigational Product 31
9.3. Treatment After the End of the Study 31

10. ADVERSE EVENTS (AE) AND SERIOUS ADVERSE EVENTS (SAE) 32
10.1. Definition of an AE 32
10.2. Definition of a SAE 32
10.2.1. Clinical Laboratory Abnormalities and Other Abnormal Assessments as AEs and SAEs 33
10.3. Time Period, and Frequency of Detecting AEs and SAEs 33
10.4. Prompt Reporting of SAEs to GSK 34
10.4.1. Timeframes for Submitting SAE Reports to GSK 34
10.5. AE and SAE Documentation and Follow-up Procedures 34

11. DATA ANALYSIS AND STATISTICAL CONSIDERATIONS 34
11.1. Hypotheses 34
11.2. Study Design Considerations 35
11.2.1. Choice of Non-Inferiority Margin 35
11.2.2. Sample Size Assumptions 36
11.2.3. Sample Size Sensitivity 37
11.2.4. Sample Size Re-estimation 38
11.3. Data Analysis Considerations 38
11.3.1. Analysis Populations 38
11.3.2. Analysis Data Sets 39
11.3.3. Treatment Comparisons 39
11.3.3.1. Primary Comparisons of Interest 39
11.3.3.2. Other Comparisons of Interest 39
11.3.4. Interim Analysis 39
11.3.5. Key Elements of Analysis Plan 40
11.3.5.1. Data derivation 40
11.3.5.2. Missing data 40
11.3.5.3. Assessment windows 40

11.3.5.4. Methods of Analysis 41
11.3.5.5. Subgroup analyses 41
11.3.5.6. Deviations from the Planned Analyses 41
11.3.5.7. Efficacy Analyses 41
11.3.5.8. Safety Analyses 42
11.3.5.8.1. Extent of Exposure 42
11.3.5.8.2. Adverse Events 42
11.3.5.8.3. Clinical Laboratory Evaluations 43
11.3.5.8.4. G6PD Evaluations 43
11.3.5.9. Pharmacokinetic Analyses 43

12. STUDY CONDUCT CONSIDERATIONS 44
12.1. Regulatory and Ethical Considerations, Including the Informed Consent Process 44
12.2. Quality Control (Study Monitoring) 44
12.3. Quality Assurance 45
12.4. Study and Site Closure 45
12.5. Records Retention 45
12.6. Provision of Study Results and Information to Investigators 46
12.7. Data Management 46
12.8. Independent Data Monitoring Committee (IDMC) 47

13. REFERENCES 48

APPENDICES 51
Appendix 1: Time and Events Table 51
Appendix 2: Country Specific Requirements 53
Appendix 3: Dosing of study drugs 54
Appendix 4: Investigators and collaborators associated with Kintampo Health Research Centre, Ghana 55
Appendix 5: Protocol Amendment 1 changes 56
Appendix 6: Protocol Amendment 2 changes 57
Appendix 7: Protocol Amendment 3 changes 60
Appendix 8: Protocol Amendment 04 62

ABBREVIATIONS

ACPR	Adequate Clinical and Parasitological Response
ACT	Artemisinin-based Combination Therapy
AE	Adverse Event
ART	Artesunate
CCG	Chlorcycloguanil
CDA	Chlorproguanil-dapsone-artesunate
CPG	Chlorproguanil
CQ	Chloroquine
DCSI	Developmental Core Safety Information
DDS	Dapsone
DHA	Dihydroartemisinin
DHFR	Dihydrofolate reductase
DHPS	Dihydropteroate synthetase
DNA	Deoxyribonucleic acid
eCRF	Electronic Case Record Form
ETF	Early Treatment Failure
FCT	Fever Clearance Times
G6PD	Glucose 6 phosphate dehydrogenase
GCP	Good Clinical Practice
GCSP	Global Clinical Safety and Pharmacovigilance
GSK	GlaxoSmithKline
I.B.	Investigator Brochure
IDMC	Independent Data Monitoring Committee
IEC / IRB	Independent Ethics Committee / Independent Review Board
ITT	Intent to Treat
LCF	Late Clinical Failure
LPF	Late Parasitological Failure
MedDRA	Medical Dictionaries for Regulatory Activities
MMV	Medicines for Malaria Venture
MSP-1	Merozoite Surface Protein -1
MSP-2	Merozoite Surface Protein -2
NCSH	National Center for Health Statistics
PCR	Polymerase Chain Reaction
PCT	Parasite Clearance Times
P.f.	*Plasmodium falciparum*
*Pf*mdr	*P. falciparum* multi-drug resistance
PK	Pharmacokinetic
PP	Per Protocol
RAMOS	Registration And Medication Ordering System
RAP	Reporting and Analysis Plan
RBC	Red Blood Cell
SAE	Serious Adverse Events
SOC	System Organ Class
S/P	Sulphadoxine/Pyrimethamine

Abbreviations (Continued)

SRM	Study Reference Manual
SRT	Safety Review Team
WBC	White Blood Cell
WCC	White Cell Count
WHO	World Heath Organisation

Trademark Information

Trademarks of the GlaxoSmithKline group of companies
LAPDAP

Trademarks not owned by the GlaxoSmithKline group of companies
Coartem
NONMEM
Riamet

PROTOCOL SUMMARY

Rationale

LAPDAP™ (chlorproguanil-dapsone) has been approved for the treatment of uncomplicated *Plasmodium falciparum* malaria in a number of countries across sub-Sahara Africa, and by the UK's Medicines and Healthcare products Regulatory Agency.

The combination of chlorproguanil-dapsone-artesunate (CDA) is being developed to supersede LAPDAP for the same indication, but the addition of an artemisinin derivative, artesunate, should provide additional population benefits over LAPDAP alone. The artemisinins have been demonstrated to rapidly reduce parasite load and have activity against the sexual stages of the *P.falciparum* lifecycle. The addition of a second agent to the LAPDAP combination should also protect against the selection of resistant strains of *P.falciparum*.

Artemether-lumefantrine (Coartem) is the only available fixed-dose Artemisinin-based Combination Therapy actually available and is considered as the gold standard for the treatment of *P. falciparum* malaria. This study will therefore aim to demonstrate the non-inferiority of the combination of CDA to artemether-lumefantrine in terms of efficacy at 28-days. The key secondary objectives will compare the Parasite Clearance Times (PCT) and the Fever Clearance Times (FCT) between CDA and artemether-lumefantrine.

Objective(s)

Primary objective:

- To demonstrate CDA is non-inferior to artemether-lumefantrine when comparing efficacy at day 28.

Secondary objectives:

- To compare the efficacy of CDA vs artemether-lumefantrine at day 14 and 42.
- To compare the asexual parasite densities over time and the parasitological clearance time (PCT) of CDA vs artemether-lumefantrine.
- To compare the fever clearance time (FCT) of CDA vs artemether-lumefantrine.
- To compare of gametocyte densities over time of CDA vs artemether-lumefantrine.
- To report the safety and tolerability of CDA.
- To describe the population pharmacokinetic profile of CPG, CCG, DDS, ART and DHA in the CDA treatment group.

Endpoint(s)

Primary endpoint:

- Parasitological cure rate, PCR corrected, at day 28 in the PP population. The ITT population is a key supportive analysis.

Secondary endpoints:

- Parasitological cure rate, PCR-corrected, at day 14 and 42.
- ACPR, and ACPR PCR corrected at day 14, 28 and 42.
- Summary of asexual parasite densities on days 0, 1, 2, 3, 7, 14, 28 and 42 by treatment group.
- PCT and FCT by treatment group.
- Summary of gametocyte densities on days 0, 1, 2, 3, 7, 14, 28 and 42 by treatment group.
- Safety assessments, including AE and SAE reporting, laboratory results and Glucose-6-phosphate-dehydrogenase (G6PD) status.
- Population PK parameters.

Study Design

This study will be a multi-centre, parallel group, double-blind, double-dummy, randomised controlled trial of CDA vs artemether-lumefantrine. Each subject will be randomized to receive CDA or artemether-lumefantrine in a ratio of 2:1 respectively. Subjects will be screened and randomized on day 0, and admitted for days 0-3; dosed for once daily for 3 successive days (days 0, 1 and 2), seen at home by a field-worker on days 4, 5 and 6, then followed up at clinic on days 7, 14, 28 and 42, and on any additional day on request.

Study Population

One thousand three hundred and ninety five male and female subjects presenting with acute uncomplicated *P. falciparum* malaria will be recruited. Eligible subjects will be aged ≥ 12 months, and weigh ≥ 7.5kg.

Study Assessments

Study assessments include daily assessment of peripheral parasite levels, tympanic temperature measurements, assessment of clinical chemistry and haematology parameters, analysis of a subject's G6PD status, collection of PK samples for population PK analysis and collection of AE and SAE data,

1. INTRODUCTION

1.1. Background

CDA is a combination of chlorproguanil, dapsone and artesunate, being developed in a public-private partnership with the Medicines for Malaria Venture (MMV), World Health Organisation (WHO-TDR) and academic partners from the London School of Hygiene and Tropical Medicine, University of Liverpool and the Liverpool School of Tropical Medicine as a treatment for acute uncomplicated *P. falciparum* malaria.

The combination of chlorproguanil HCl (CPG) and dapsone (DDS) has previously been developed as a fixed dose combination tablet, marketed as LAPDAP™. CPG has a persistent antimalarial action mainly through the production of its major metabolite, chlorcycloguanil (CCG), acting specifically on *P. falciparum* dihydrofolate reductase enzyme (DHFR). DDS inhibits the dihydropteroate synthetase enzyme (DHPS) in malarial species resulting in inhibition of folate metabolism.

Artesunate is a semi synthetic derivative of an extract of *Artemisia annua*. The artemisinin group of drugs are rapidly acting and potent antimalarials; their mechanism of action is thought to be through the generation of free radicals, which interfere with the parasite's ability to neutralise haem.

Different metabolic pathways metabolise the three components of CDA and none of the components has a potent inhibition potential for the major metabolic pathways of the other components. These data taken together suggest that the likelihood of a significant drug interaction between them in combination is unlikely. Neither have any of the components demonstrated hepatic enzyme induction or inhibition properties that would raise serious concerns when considering their potential for interacting adversely with other drugs.

The different components of CDA all interact with red blood cells, and the parasites within them. It has previously been demonstrated that haemoglobin levels of subjects treated with LAPDAP are reduced at day 7 [Alloueche, 2004]. Artesunate has been shown to reduce reticulocyte levels at 96-hours post dose in healthy volunteers, in study SB-714703/001. The addition of artesunate to LAPDAP could therefore theoretically lengthen the time to recover from the reduction in haemoglobin, due to a suppression of reticulocyte production, however, probably because of the fast action of artesunate on the parasite kill rate, this was not observed in the small number of subjects treated with both LAPDAP and artesunate in the phase II dose-ranging study (SB-714703/003).

The main scientific objective of the CDA development process is to obtain a safe, effective and affordable Artemisinin-based Combination Therapy (ACT) for the treatment of uncomplicated *P. falciparum* malaria, especially in Africa.

The burden of malaria is mainly carried by tropical Africa which has 90% of the global incidence and approximately two million deaths annually. Almost everyone in this region becomes infected during childhood, and most morbidity/mortality is seen in children

under the age of 5 years. Morbidity and mortality are also seen in young childbearing women who, although ‘partially immune’, are at risk from severe anaemia during pregnancy and may bear low birth-weight babies.

Chloroquine (CQ) was the treatment of choice for most of Africa for many years, but resistance is now widespread. Sulphadoxine/pyrimethamine (S/P) was introduced into a number of countries as a low-cost alternative to chloroquine, however resistance was quick to develop to S/P in sub-Saharan Africa (SSA), most likely due to the long terminal half-life of the component drugs. Both S/P and CQ are being phased out of use as monotherapy in favour of ACT, such as artemether-lumefantrine, or other combinations of currently available antimalarials, in accordance with the recommendations of the World Health Organisation - Roll Back Malaria Partnership.

Usage of artemether-lumefantrine is currently restricted because of its price, and the limited availability of the drug. The use of other loose combination ACTs is not always ideal either, as the non- artemisinin drug in these combinations often has existing underlying resistance e.g S/P and amodiaquine. There is therefore an unmet clinical need for a cost effective malaria treatment with a low propensity for drug resistance.

Data from the CDA phase II dose-ranging study, SB-714703/003, has demonstrated some of the advantages of adding artesunate to LAPDAP, like shorter parasite and fever clearance times and a reduction in gametocyte load. Based on the results of this phase II study, the decision was made to proceed to the phase III programme of the fixed-dose combination tablets of CDA with a target artesunate dose of 4 mg/kg/day.

The phase III programme for CDA consists of two phase III studies. This study will compare CDA to artemether-lumefantrine, and a second study (SB-714703/006) will compare CDA to LAPDAP. The main objective of these studies will be to determine the efficacy of CDA using a 28-day parasitological cure rate, PCR corrected, as primary endpoint. Assessment of parasitological cure rate at 28 days is appropriate, based on the pharmacokinetic properties of the component drugs of CDA [White, 2002]. Haematological indices will be also be monitored closely in both these studies.

1.2. Rationale

The combination CPG and DDS as LAPDAP has already been shown to be efficacious against *P.falciparum* in adults and children in sub-Sahara Africa. The addition of artesunate to LAPDAP has been demonstrated to increase the parasite kill rate and reduce the chance of any parasites escaping treatment over the 3-day course. The addition of artesunate is also anticipated to have the population benefit of protection against the development of resistant strains of *P.falciparum*, although it will not be possible to demonstrate this in a clinical trial. One further population benefit of the artemisinin drugs are their ability to suppress the sexual forms of the parasite (gametocytes), which should reduce infectivity after antimalarial treatment and potentially lower transmission rates with widespread use, including the spread of any parasites resistant to the partner drug.

The aims of this phase III study are to compare the efficacy of a fixed dose combination tablet of CDA to artemether-lumefantrine, and collect supporting safety and population pharmacokinetic data. This will be a multi-centre, double-blind, double-dummy, randomised trial in children up to and including 14 years of age. A similar study with LAPDAP as comparator will also be conducted (SB-714703/006).

Artemether-lumefantrine is a combination anti-malarial of artemether and lumefantrine, sold as Coartem or Riamet (Novartis). The t1/2 of artemether is 2-3 hours. Artemether is converted to dihydroartemisinin (DHA), which is the same active metabolite as artesunate. The t1/2 of lumefantrine is 2-3 days in healthy volunteers, and 5-10 days in patients with uncomplicated *P.falciparum* malaria. Artemether-lumefantrine is the only currently available fixed-formulation artemisinin combination therapy available for malaria treatment, and is therefore considered the gold standard for comparison.

2. OBJECTIVE(S)

2.1. Primary

- To demonstrate CDA is non-inferior to artemether-lumefantrine when comparing efficacy at day 28.

2.2. Secondary

- Comparison of efficacy of CDA vs artemether-lumefantrine at day 14 and 42.
- Comparison of asexual parasite densities over time and parasite clearance time (PCT) of CDA vs artemether-lumefantrine.
- Comparison of fever clearance time (FCT) of CDA vs artemether-lumefantrine.
- Comparison of gametocyte densities over time.
- To report the safety and tolerability of CDA.
- To describe the population pharmacokinetic profile of CPG, CCG, DDS, ART and DHA in the CDA treatment group.

3. ENDPOINT(S)

3.1. Primary

Primary Efficacy Endpoint:

- Parasitological cure rate, PCR-corrected, at day 28, in the per-protocol population. Parasitological cure rate is defined as the clearance of the initial malaria infection by day 7 and remaining free of this infection to the day of assessment (PCR of the parasite genotype is used to distinguish between a new malaria infection and a re-appearance of the initial infection (recrudescence)). The intent-to-treat population is a key supportive analysis.

3.2. Secondary

Secondary Efficacy Endpoints:

- Parasitological cure rate, PCR-corrected, at day 14 and 42, by treatment group.
- ACPR, and ACPR PCR corrected at day 14, 28 and 42, by treatment group.
- Summary of asexual parasite densities on days 0, 1, 2, 3, 7, 14, 28 and 42 by treatment group.
- PCT by treatment group.
- FCT by treatment group.
- Summary of gametocyte densities on days 0, 1, 2, 3, 7, 14, 28 and 42 by treatment group.

Safety endpoints:

- Adverse events (AEs) and serious adverse events (SAEs) reported by treatment group.
- Laboratory tests (including parameters relating to anaemia and haemolytic anaemia), by treatment group.
- Glucose-6-phosphate-dehydrogenase (G6PD) status, as determined by genotype and phenotype testing, by treatment group.

Pharmacokinetic endpoints:

- Population PK parameters e.g. CL/F, V/F, ka for CPG, CCG, DDS, ART and DHA.

4. STUDY DESIGN

- Parallel treatment groups.
- Active comparator (artemether-lumefantrine).
- Randomised allocation at time of enrolment.
- Treatment allocation of 2:1 (CDA:artemether-lumefantrine).
- Subjects will be randomised to one of two treatment groups:
    a. Active CDA as a fixed combination tablet once daily on days 0, 1 and 2, plus placebo artemether-lumefantrine twice daily on days 0, 1, 2, or
    b. Placebo CDA once daily on days 0, 1 and 2, plus active artemether-lumefantrine twice daily on days 0, 1, 2.
- Double-blind, double-dummy.
- Multi-centre across Sub-Sahara Africa.
- An Independent Data Monitoring Committee (IDMC) will be convened to oversee both this study and study SB-714703/006.

- In-patient study to oversee dosing (artemether-lumefantrine - twice daily for 3 days).
- Screening, recruitment, treatment allocation - day 0.
- Dosing days 0, 1, 2, hospitalised until day 3.
- Home visits by a field-worker days 4, 5, 6.
- Follow-up days 7, 14, 28 and 42.

5. STUDY POPULATION

5.1. Number of Subjects

1395 subjects (930 CDA: 465 artemether-lumefantrine) will be randomised, allowing for a 30% withdrawal / loss to follow-up anticipated for a 28-day follow-up assessment based on experience from the previous SB-714703/003 study, to yield 975 evaluable subjects for the primary efficacy analysis (650 CDA: 325 artemether-lumefantrine).

5.2. Eligibility Criteria

5.2.1. Inclusion Criteria

A subject will be eligible for inclusion in this study only if all of the following criteria apply:

1. Acute, uncomplicated *P.falciparum* malaria, microscopically confirmed infection of 2,000 – 200,000 parasites / uL.
2. Tympanic temperature at screening of ≥37.5°C or confirmed history of fever within previous 24-hours.
3. Male or female child.
4. Aged ≥12months, up to and including 14 years old.
5. Weigh ≥7.5kg.
6. Screening haemoglobin of ≥7g/dl, or haematocrit of ≥25% (If Hb not available at screening).
7. Willingness to comply with the study visits and procedures, as outlined in the informed consent form.
8. Written or oral witnessed consent has been obtained from parent or guardian.
9. Assent is given by a child aged ≥12 years, in addition to the consent of their parent or guardian.

5.2.2. Exclusion Criteria

A subject will not be eligible for inclusion in this study if any of the following criteria apply:

1. Features of severe/complicated falciparum malaria.
2. Hypersensitivity to active substances (chlorproguanil, dapsone, artesunate, artemether, lumefantrine), or excipients of the investigational products.
3. Known allergy to biguanides, sulphones, sulphonamides, artemisinin derived products or aminoalcohol drugs.
4. Known history of G6PD deficiency, methaemoglobin reductase deficiency, haemoglobin M or E, or porphyria.
5. Infants with a history of hyperbilirubinaemia during the neonatal period.
6. Use of concomitant medications that may induce haemolysis or haemolytic anaemia from the WHO list of essential drugs.
7. Evidence of any concomitant infection at the time of presentation (including *P. vivax, P. ovale* and *P. malariae*).
8. Any other underlying disease that may compromise the diagnosis and the evaluation of the response to the study medication (including clinical symptoms of immunosuppression, tuberculosis, bacterial infection; cardiac or pulmonary disease).
9. Malnutrition, defined as a child whose weight-for-height is below -3 standard deviations or less than 70% of the median of the NCHS/WHO normalised reference values.
10. Treatment within the past three months with mefloquine or mefloquine-sulphadoxine-pyrimethamine; twenty-eight days with sulphadoxine/pyrimethamine, sulfalene/pyrimethamine, , lumefantrine or artemether/lumefantrine, amodiaquine, atovaquone or atovoquone/proguanil, halofantrine; 14-days with chlorproguanil/dapsone, or 7-days with quinine (full course), proguanil, artemisinins, tetracycline doxycycline or clindamycin.
11. Unknown antimalarial drug use within the past 28 days.
12. Use of an investigational drug within 30 days or 5 half-lives whichever is the longer.
13. Previous participation in this study.
14. Female subjects of child-bearing age, who have had a positive pregnancy test at screening, or do not give their consent to take a pregnancy test.
15. Female subjects who will be breast-feeding an infant for the duration of the study.

5.2.3. Other Eligibility Criteria Considerations

To assess any potential impact on subject eligibility with regard to safety, the investigator must refer to the following document(s) for detailed information regarding warnings, precautions, contraindications, adverse events, and other significant data pertaining to the investigational product(s) being used in this study: CDA Investigator Brochure, LAPDAP product label, Coartem product label.

6. STUDY ASSESSMENTS AND PROCEDURES

6.1. Demographic and Baseline Assessments

Screening:

- Clinical assessment.
- Microscope blood slide prepared for asexual parasite count, plus gametocyte count (See Section 6.3.1. and Study Reference Manual for further details).
- Filter paper prepared to collect baseline parasite DNA (MSP-1 and MSP-2 markers) (See Section 6.3.2).
- Tympanic temperature measured.
- Weight (kg) and age (date of birth), height or length recorded to calculate the malnutrition status for eligibility.
- Haemoglobin or haematocrit result; this blood sample can be used to analyse the remaining day 0 haematology values too.
- Informed consent process for all subjects.
- Prior and current medical history recorded.
- Current medications reviewed, including anti-malarial drug use in previous 3-months.
- Urine test for chloroquine, all subjects (see Study Reference Manual).
- Pregnancy testing on female children over the age of 12 years, or if menstruation has commenced earlier (serum test day 0), see Section 6.2.4 for definition.
- Data on previous malaria episodes in children aged ≤2years will be recorded in the eCRF.

6.2. Safety

6.2.1. Clinical assessment and vital signs

Clinical assessment, daily whilst an in-patient, on each scheduled follow-up visit at day 7, 14 28 and 42, and on any additional unscheduled visits.

Tympanic temperature measurements on day 0 at screening and pre-dose & every 8hours during the in-patient stay until discharge on day 3; single assessments on days 3, 7, 14, 28 and 42.

Subjects should be able to return to the clinic for unscheduled visits at any time during the 42-day study period.

6.2.2. Haematology and clinical chemistry

Due to the location of the study sites, the transit time required for shipment of samples and logistical constraints of cold-chain storage, haematology and clinical chemistry tests will be conducted by local laboratories for this study, rather than a central laboratory.

Clinical chemistry blood samples will be taken on days 0 (pre-dose), 3, 7 and 42. Samples should only be taken on day 14 or 28 if the previous results were abnormal. Clinical chemistry tests may be performed at the physician's discretion at any time on unscheduled follow-up visits.

Clinical chemistry tests to include: serum creatinine, serum total and indirect bilirubin, serum aspartate aminotransferase (AST) and serum alanine aminotransferase (ALT).

Haematology blood samples will be taken on days 0 (screening), 1, 2, 3, 7, 14, 28 and 42. Samples taken on days 0, 1 and 2 should be taken **before** dosing. Haematology tests may be performed at the physician's discretion at any time on unscheduled follow-up visits. Haematology tests to include: haemoglobin, haematocrit, reticulocyte count (% of red blood cells and absolute count calculated against red blood cells), red blood cell count, white blood cell count, platelet count, methaemoglobin when available.

6.2.3. G6PD analysis

Two drops of blood will be collected onto pre-printed filter papers on day 0, prior to administration of the first dose of study medication, for subsequent DNA extraction and PCR of the subject's G6PD gene. Loci of interest in the G6PD A- deficient variant most commonly found in sub-Sahara Africa are: 202A,376G; 376G,680A and 376G,968C. The Santamaria variant (376G,542T) is also found in the extreme West Africa and in The Gambia.

An aliquot of the day 0 haematology whole blood sample will be used to test for G6PD deficiency using the NADPH fluorescence method. Blood may be stored at 4°C for up to one week prior to analysis. A commercially available test will be used, run against standard controls (G6PD normal, G6PD deficient and G6PD intermediate). Filter papers

will be stored at 4°C in the dark until analysis, see Study Reference Manual for more detail.

A repeat phenotype test will be conducted at day 28 on any subject who experiences a fall in haemoglobin of ≥40% from baseline during the study, or if their day 0 reticulocyte count was high.

6.2.4. Pregnancy

Female subjects of child-bearing potential, defined as aged ≥12 years will be asked to take a serum pregnancy test prior to enrolment into the study. They will be asked to take a urine pregnancy test on day 42 or on early withdrawal from study.

Female subjects of child-bearing potential, as defined as aged ≥12 years, and sexually active should use barrier contraceptive measures for the duration of the study period. Condoms and spermicide will be provided by the investigator / study team, and appropriate counselling should be given to female subjects of child-bearing potential about the risks of becoming pregnant and exposing the foetus to study drugs during the consent process. See Study Reference Manual for further details.

6.2.4.1. Time period for collecting pregnancy information

Pregnancies will be reported from the time of the first dose to the end of follow-up (day 42, unless the subject is withdrawn earlier).

6.2.4.2. Action to be taken if pregnancy occurs

The investigator will collect pregnancy information on any female subject, who becomes pregnant while participating in this study. The investigator will record pregnancy information on the appropriate form and submit it to GSK within 2 weeks of learning of a subject's pregnancy. The subject will also be followed to determine the outcome of the pregnancy. Information on the status of the mother and child will be forwarded to GSK. Generally, follow-up will be no longer than 6 to 8 weeks following the estimated delivery date. Any premature termination of the pregnancy will be reported.

While pregnancy itself is not considered to be an AE or SAE, any pregnancy complication or elective termination of a pregnancy for medical reasons will be recorded as an AE or SAE (see AE/SAE section of the protocol and the SRM for definitions and a description of follow-up).

A spontaneous abortion is always considered to be an SAE and will be reported as such. Furthermore, any SAE occurring as a result of a post-study pregnancy and is considered reasonably related to the investigational product by the investigator, will be reported to GSK as described in section entitled, "Post-study AEs and SAEs" of the SRM. While the investigator is not obligated to actively seek this information in former study participants, he or she may learn of an SAE through spontaneous reporting.

6.2.5. Home visits

A field-worker should make a home visit to all study participants on days 4, 5 and 6 to ensure that the subject is well enough to remain at home. If any subject is found to be unwell, they should be transported to the study site and an unscheduled visit conducted.

6.3. Efficacy

6.3.1. Parasitology

Asexual parasite counts:

Microscope blood slides will be prepared on day 0 at screening and pre-dose, then every 8-hours during the in-patient stay until discharge on day 3. Microscope blood slides will be prepared at each subsequent visit on days 7, 14, 28 and 42. At each timepoint two thick and one thin film should be prepared. See Study Reference Manual for staining and counting methodology.

At screening the subject's parasitaemia should be calculated against a nominal white blood cell count (WCC) of 8,000/ul, as the haematology result will not be available at this stage:

Screening parasitaemia /ul = (number of parasites / number of WBCs counted) x 8,000

The day 0 pre-dose parasitaemia should be calculated against the subject's day 0 WCC value to reflect the baseline parasitaemia, just prior to dosing. All subsequent parasitaemias should be calculated against the WCC for that visit e.g. on day 3:

d3 parasitaemia /ul = (number of parasites / number of WBCs counted) x d3 WCC

If the thick film contains >10 asexual parasites/ul, the thin film should be read and parasites counted against a minimum of 500 red blood cells. Parasitaemia from thin films is calculated as:

Parasitaemia/ul = (number of parasites / number of RBCs counted) x RBC count

The calculated parasitaemia/ul will be recorded into the eCRF for each timepoint. See the Study Reference Manual for quality control procedures.

Gametocyte counts:

Thick film slides will be read for gametocytes on day 0 (screening and pre-dose), 1, 2, 3, 7, 14, 28 and 42. If gametocytes are present, they will be counted against a minimum of 200 WBCs and their density calculated as:

Gametocytes /ul = (number of gametocytes / number of WBCs counted) x WCC

6.3.2. Parasite DNA analysis

Two drops of peripheral blood will be collected onto pre-printed filter papers, for subsequent DNA extraction and PCR analysis of *P.falciparum* DNA, on all subjects at screening and any day on or after day 7 when a blood slide is prepared.

PCR of the *P.falciparum* genes MSP-1, MSP-2 and GLURP will be used to distinguish between the initial infection reappearing (recrudescence or inadequate therapy received) and a new infection occurring on any day on or after day 7 in the 42-day follow-up period.

Resistance marker work will investigate the presence of mutations associated with resistance of anti-folate drugs in the genes encoding DHFR and DHPS [Kublin, 2002] and resistance to lumefantrine, *Pf*mdr gene, as below. DNA extraction from the dried filter-paper blood spots and mutation-specific nested PCR and /or restriction digestions to detect these mutations will be performed at a single laboratory, see Study Reference Manual for further details.

DHFR codons 51, 59, 108 and 164; DHPS codons 437, 540, 581 and 613; *Pf*mdr186N

6.3.3. Parasitological Cure Rate

Whether a subject has achieved the primary endpoint of parasitological cure rate will be determined based on the parasitology data with PCR correction for re-infection / recrudescence as described above on day 28, and on day 14 and 42 for the secondary endpoint analysis. If a subject has a recrudescence of the initial infection, or the PCR result is inconclusive, they will be categorized as a failure. Subjects without parasitology data on day 14, 28 or 42 will be considered not assessable for that timepoint.

6.3.4. Adequate Clinical and Parasitological Response

Subjects will also be assessed for this secondary endpoint against the following definitions and described as either having an Adequate Clinical Parasitological Response (ACPR), early treatment failure, late parasitological failure, late clinical failure, or being not assessable, on Days 14, 28 and 42

Early Treatment Failure (ETF):

- Development of danger signs or severe malaria on Day 1, 2 or 3 (as defined in World Health Organisation. Severe falciparum malaria. *Trans R Soc Trop Med Hyg,* 2000; 94, supplement 1, [World Health Organisation, 2000]) in the presence of parasitaemia
- Parasitaemia on Day 2 higher than Day 0 count, irrespective of tympanic temperature
- Parasitaemia on Day 3 with tympanic temperature ≥ 38.0°C
- Parasitaemia on Day 3 ≥ 25% of count on Day 0

Late Clinical Failure (LCF):

- Development of danger signs or severe malaria after Day 3 in the presence of parasitaemia, without previously meeting any of the criteria of ETF
- Presence of parasitaemia and tympanic temperature ≥ 38.0°C (or history of fever) on any day from Day 4 to Day 28, without previously meeting any of the criteria of ETF

Late Parasitological Failure (LPF):

- Presence of parasitaemia on any day from Day 7 to Day 28 and tympanic temperature < 38.0°C, without previously meeting any of the criteria of ETF or LCF

Adequate Clinical and Parasitological Response (ACPR):

- Absence of parasitaemia on Day 28 irrespective of tympanic temperature without previously meeting any of the criteria of ETF, LCF or LPF

Treatment of ETF, LCF or LPF:

Subjects meeting the criteria for either ETF, LCF or LPF will receive treatment with appropriate rescue medication, as determined by each individual site, and will be followed up for safety assessment until day 42, or until resolution of the malaria episode if this persists for longer than day 42.

6.3.5. Clearance times (PCT and FCT)

Subjects will also be assessed for these secondary endpoints against the following definitions:

- **PCT**: Time needed to clear asexual parasite forms from the blood, parasite numbers fall below the limit of detection in a thick blood smear and remain undetectable for at least 48 hours.
- **FCT:** Time from the first dose of treatment to the time when body temperature falls to normal and remains so for at least 48 hours.

6.4. Urine testing for antimalarial drugs

A urine sample will be taken for all subjects to test for the presence of chloroquine.

6.5. Pharmacokinetics

Up to five blood samples for population PK analysis will be collected from each subject. These samples will be collected within varying time windows:

1. 0.25-0.5 h
2. 1-3 h
3. 4-8 h
4. 12-24 h
5. Day 7

Samples 1-4 can be taken after any of the morning doses on Days 0-2. The fifth sample can be taken any time on Day 7. Sampling times within a particular window should be spread across that window among subjects rather than being grouped at extreme ends of the window (e.g. 8-24 h window – not all samples be taken at 8 h or 24 h). The dates and exact times of sampling, as well as the dates and exact times of drug administration must be recorded in the eCRF.

Method for handling PK blood samples

Blood, approximately 2mL, will be collected into polypropylene tubes containing lithium heparin, mixed gently and placed on crushed wet-ice for no longer than 30mins, until centrifugation at 1800g for 10 minutes using a refrigerated centrifuge. The resultant plasma will be separated, and transferred in equal volumes to two uniquely labelled clear polypropylene tubes and frozen immediately over solid carbon dioxide or in a freezer at nominal –80°C. Samples will be transported frozen to WW Bioanalysis, DMPK, Ware, to be stored at approximately -80°C until analysed. Plasma concentrations of CPG, CCP, DDS, ART and DHA will be determined using currently approved methods by the WW Bioanalysis group, Ware and be used to evaluate the population pharmacokinetics of these compounds, if data permit.

7. INVESTIGATIONAL PRODUCT(S)

7.1. Description of Investigational Product

Chloroproguanil/Dapsone/Artesunate Tablets (CDA Tablets) are available as brown or yellow, peanut shaped, film-coated tablets. These contain either 12/15/24 mg or 60/75/120 chlorproguanil (as the hydrochloride)/Dapsone/Artesunate respectively. Matching placebo tablets to both tablets strengths are also available.

Artemether /lumefantrine Tablets are available as pale yellow uncoated tablet. These tablets will be coloured film-coated to differentiate them from the commercial product These contain 20/120 mg Arthemether/Lumefantrine respectively. Matching placebo tablets are also available.

7.2. Dosage and Administration

Target doses of CPG, DDS and artesunate will be 2, 2.5 and 4 mg/kg/day respectively. Treatment will be administered once daily for 3 days, dosed according to weight. CDA tablets will contain a break-line and are intended to be broken in half in some weight bands to facilitate dosing. See Appendix 1 for dosing schedule.

Artemether-lumefantrine will be given as a 6-dose course, administered twice daily for 3 days, with the second dose administered 8 hours after the initial dose. Artemether-lumefantrine is also dosed according to weight (See Appendix 1 for dosing schedule). Artemether-lumefantrine is recommended to be administered with breast milk, or condensed milk in children, therefore both treatment groups will receive their study medication in this manner.

Breast milk or at least 5ml condensed milk should be given at least 30 mins prior to administration of study drug.

Younger children may receive their tablets crushed and mixed with water just prior to administration for both treatment groups. It is intended to record whether tablets are crushed in the electronic case report form (eCRF).

Subjects who vomit any dose of study medication within the first 30mins after dosing will be re-dosed. Subjects who vomit the second dose within the next 30mins will be given appropriate rescue medication and should remain in the study for safety assessment until day 42.

7.3. Dose Rationale

The target doses of CPG and DDS are equivalent to the marketed product LAPDAP, 2mg/kg/day CPG, 2.5mg/kg/day DDS. The artesunate dose to combine with CPG and DDS was determined from a dose-ranging study (SB-714703/003); 4 mg/kg/day, as discussed in the Introduction and Investigator's Brochure.

The 6-dose course for artemether-lumefantrine was chosen as this has been shown to be superior to the 4-dose course and it is anticipated that the 6-dose course will be in mainstream use in SSA by the time CDA is projected to be available on the market. Comparing CDA to the 4-dose course would be open to criticism that we were comparing our drug against an inferior product.

7.4. Blinding

CDA tablets and matching placebo, as well as commercial Coartem (artemether-lumefantrine) tablets and matching placebo (colour film-coated to differentiate the active and placebo tablets from the commercial product) will be used in the study.

Treatment allocation will be assigned via the Registration and Medical Ordering System (RAMOS).

Only in the case of an emergency, when knowledge of the investigational product is essential for the clinical management or welfare of the subject, the investigator may unblind a subject's treatment assignment. The investigator will, whenever possible, discuss options with the Medical Monitor, on-call physician, or appropriate GSK study personnel before unblinding. If the blind is broken for any reason and the investigator is unable to contact GSK prior to unblinding, the investigator must notify GSK **as soon as possible following** the unblinding incident **without revealing the subject's study treatment assignment,** unless the information is important to the safety of subjects remaining in the study. In addition, the investigator will record the date and reason for revealing the blinded treatment assignment for that subject in the appropriate data collection tool (as defined in Section 12.7).

If the treatment blind needs to be broken for an individual subject during the study, as a result of a medical emergency, it will be done by placing a call to RAMOS to break the blind. Further details will be provided in the study reference manual. If the blind is broken for a subject, they should remain in the study for safety assessment, but will be ineligible for the per-protocol population.

If a serious adverse event (SAE; as defined in Section 10.2, "Definition of an SAE") is reported to GSK, Global Clinical Safety and Pharmacovigilance (GCSP) staff may unblind the treatment assignment for the individual subject. If an expedited regulatory report to one or more regulatory agencies is required, the report will identify the subject's treatment assignment. When applicable, a copy of the regulatory report may be sent to investigators in accordance with relevant regulations, GSK policy, or both.

7.5. Treatment Assignment

Subjects will be assigned to study treatment in accordance with the randomisation schedule. The randomisation schedule will utilise centre-based allocation.

The randomisation schedule will be generated by the Biomedical Data Sciences group at GSK, using Randall software. RAMOS will be used to allocate subjects to the treatment groups.

Once a randomisation number has been allocated to a subject, it cannot be re-assigned to any other subject at the site.

7.6. Packaging and Labelling

The investigational products will be supplied as individual bottles containing sufficient tablets for the whole dosing period of the study, with two bottles being allocated per patient. One bottle will contain either CDA active or placebo (either 12:15:24 or 60:75:120 tablet size), and the other artemether-lumefantrine active or placebo. Each bottle will be labelled with the following information; drug product (including fill count), storage conditions and dosage directions.

The contents of the label will be in accordance with all applicable regulatory requirements.

7.7. Preparation

The only preparation that may be applicable to either treatment would be the crushing of the tablets prior to administration in younger children.

Tablets will be crushed in a pestle and mortar, mixed with a little water for immediate administration to the younger children.

7.8. Handling and Storage

Investigational product must be dispensed or administered according to procedures described herein. Only subjects enrolled in the study may receive investigational product, in accordance with all applicable regulatory requirements. Only authorized site staff may supply or administer investigational product. All investigational products must be stored in a secure area with access limited to the investigator and authorized site staff and under physical conditions that are consistent with investigational product-specific requirements.

Study drugs will be administered by the investigator, or an appropriately qualified designee.

Drugs will be kept in secure storage conditions, at a temperature of less than 30°C. Final disposition of unused investigational product will be either destroyed at site, if appropriate facilities are available, or will be returned to GSK or a third party contractor.

7.9. Product Accountability

The investigator, institution, or the head of the medical institution (where applicable) is responsible for investigational product accountability, reconciliation, and record maintenance. In accordance with all applicable regulatory requirements, the investigator or the head of the medical institution (where applicable), or designated site staff (e.g., storage manager, where applicable) must maintain investigational product accountability records throughout the course of the study. The responsible person(s) will document the amount of investigational product received from and returned to GSK (when applicable), the amount supplied and/or administered to and returned by subjects, if applicable.

Investigational product will be accounted for to the number of tablets per patient pack.

7.10. Assessment of Compliance

Compliance will be reported as adherence with treatment group allocated, and with the correct dosage for their weight. A patient will be considered to be non-compliant if they do not receive the complete 3-day treatment course of investigational product. If a subject vomits a dose of study drug, they will be considered compliant if they are redosed within 30mins, and do not vomit the re-dose.

7.11. Treatment of Investigational Product Overdose

CDA

Since chlorproguanil/dapsone has been shown to induce methaemoglobinaemia in clinical trials, clinically significant levels may be encountered in overdose. In addition to the undesirable effects seen with the recommended doses described in the DCSI [Investigator's Brochure GlaxoSmithKline Document Number GM2002/00293/01], (Section 6), the following additional events have been reported in association with dapsone or proguanil. Chlorproguanil is a dihalogenated congener of proguanil; undesirable effects associated with proguanil overdose were used in the absence of available chlorproguanil data. Coma, anxiety, mental disturbance, restlessness, hyperexcitability followed by depression, spasms or convulsions, hypotonia, hyporeflexia and ataxia, cyanosis, anuria and haematuria, sweating, fever, acute renal failure, permanent retinal damage, blindness, motor neuropathy, renal irritation and epigastric discomfort.

Treatment

The following treatments have been reported in the management of dapsone overdosage. No data was available for proguanil and symptoms should be treated as they arise. In case of accidental over-dosage, immediate induction of emesis and/or gastric lavage is recommended. Activated charcoal administered orally may be useful in shortening the half life of dapsone and monoacetyldapsone. Intravenous fluids may be required to promote diuresis. Methaemoglobinaemia may be treated with intravenous methylene blue. Methylene blue may exacerbate dapsone-induced Heinz body haemolytic anaemia and should not be given to individuals with G6PD deficiency. Ascorbic acid administration (0.5-2g) may also be of value in treating methaemoglobinaemia in G6PD deficient patients. It should not normally be used alone due to its slow speed of action. Additional supportive therapy may include oxygen, exchange transfusion and packed cell administration.

Artemether/lumefantrine

No particular risk associated with an over dosage of arthemeter/lumefantrine has been identified to-date by Novartis. In case of accidental over-dosage, routine supportive treatment including maintenance of a clear airway and adequate fluid intake should be provided.

7.12. Occupational Safety

Investigational product is not expected to pose significant occupational safety risk to site staff under normal conditions of use and administration. A Material Safety Data Sheet (MSDS)/equivalent document describing occupational hazards and recommended handling precautions either will be provided to the investigator, where this is required by local laws, or is available upon request from GSK.

However, precautions are to be taken to avoid direct skin contact, eye contact, and generating aerosols or mists. In the case of unintentional occupational exposure notify the monitor.

Precaution will be taken to avoid direct contact with the investigational product. A Material Safety Data Sheet (MSDS) describing occupational hazards and recommended handling precautions will be provided to the investigator.

8. CONCOMITANT MEDICATIONS AND NON-DRUG THERAPIES

8.1. Permitted Medications

All subjects can be given paracetamol during the study, at the discretion of the treating physician. Allowable antibiotics are penicillin, cephalosporins and aminoglycosides.

All concomitant medications taken during the study will be recorded in the eCRF. The minimum requirement is that drug name and the dates of administration are to be recorded.

8.2. Prohibited Medications

Drugs from the WHO list of essential drugs known to induce haemolysis or haemolytic anaemia (see Study Reference Manual for details). Any antimalarial, or antibiotic with antimalarial activity (erythromycin and other macrolides, co-trimoxazole and other sulphonamides, any tetracycline (including doxycycline, and quinolones.

Chloroquine: Prior treatment with chloroquine will not be considered an exclusion criterion, however, the intercurrent use of chloroquine during the study period will be a protocol violation and cause exclusion from the per-protocol population.

8.3. Non-Drug Therapies

The use of herbal remedies during the course of the study should be avoided and subjects should be encouraged to return to the study clinic for treatment if they are feeling unwell in the first instance. However, if any herbal remedies are taken during the study this should be captured in the eCRF, under concomitant medication.

9. SUBJECT COMPLETION AND WITHDRAWAL

9.1. Subject Completion

A subject will be considered to have completed the study if they have received the full course of study medication and have attended all efficacy visits.

9.2. Subject Withdrawal

A withdrawn subject is any subject who enters the study (i.e. gives informed consent), and is randomised to treatment, but does not complete the study (whether or not the subject received study medication).

9.2.1. Subject Withdrawal from Study

A subject may be withdrawn from the study any of the following reasons:

- Adverse Event
- Lost to follow-up
- Protocol violation
- Withdrawal of consent by a subject

If a subject is withdrawn from the study the following assessments should be conducted: clinical assessment, measure temperature, take haematology and clinical chemistry samples and prepare a microscope slide, plus filter paper for *P.f.* PCR any day on or after day 7. Where possible, female subjects of child-bearing potential should be asked to take a urine pregnancy test on early withdrawal from the study.

The reason for withdrawal will be recorded in the Study Conclusion form of the eCRF.

Subjects who are withdrawn will not be replaced. The sample size has been adjusted for to allow for a generous withdrawal rate and loss-to-follow-up to 28 days, see Section 11.2.1.

9.2.2. Subject Withdrawal from Investigational Product

Subjects who withdraw from the study prior to completing the full course of study medication will be given an appropriate rescue medication. The reason for withdrawal from medication will be recorded in the Investigational Product Discontinuation form of the eCRF.

Subjects withdrawing from investigational product should remain in the study for safety assessment through to day 42.

9.3. Treatment After the End of the Study

At the end of the study on the 42-day follow-up visit, if the subject is diagnosed with malaria, they will receive an appropriate rescue mediation, and be followed-up until resolution of that infection.

10. ADVERSE EVENTS (AE) AND SERIOUS ADVERSE EVENTS (SAE)

The investigator is responsible for the detection and documentation of events meeting the criteria and definition of an AE or SAE, as provided in this protocol. During the study when there is a safety evaluation, the investigator or site staff will be responsible for detecting, documenting and reporting AEs and SAEs, as detailed in both this section of the protocol and in the AE/SAE section of the SRM.

10.1. Definition of an AE

Any untoward medical occurrence in a patient or clinical investigation subject, temporally associated with the use of a medicinal product, whether or not considered related to the medicinal product.

Note: An AE can therefore be any unfavorable and unintended sign (including an abnormal laboratory finding), symptom, or disease (new or exacerbated) temporally associated with the use of a medicinal product. For marketed medicinal products, this also includes failure to produce expected benefits (i.e. lack of efficacy), abuse or misuse.

10.2. Definition of a SAE

A serious adverse event is any untoward medical occurrence that, at any dose:

a. Results in death.

b. Is life-threatening.

   NOTE: The term 'life-threatening' in the definition of 'serious' refers to an event in which the subject was at risk of death at the time of the event. It does not refer to an event, which hypothetically might have caused death, if it were more severe.

c. Requires hospitalization or prolongation of existing hospitalization.

   NOTE: In general, hospitalization signifies that the subject has been detained (usually involving at least an overnight stay) at the hospital or emergency ward for observation and/or treatment that would not have been appropriate in the physician’s office or out-patient setting. Complications that occur during hospitalization are AEs. If a complication prolongs hospitalization or fulfils any other serious criteria, the event is serious. When in doubt as to whether “hospitalization” occurred or was necessary, the AE should be considered serious.

   Hospitalization for elective treatment of a pre-existing condition that did not worsen from baseline is not considered an AE.

d. Results in disability/incapacity, or

   NOTE: The term disability means a substantial disruption of a person’s ability to conduct normal life functions. This definition is not intended to include experiences of relatively minor medical significance such as uncomplicated headache, nausea, vomiting, diarrhea, influenza, and accidental trauma (e.g. sprained ankle) which may

interfere or prevent everyday life functions but do not constitute a substantial disruption.

e. Is a congenital anomaly/birth defect.

f. Medical or scientific judgement should be exercised in deciding whether reporting is appropriate in other situations, such as important medical events that may not be immediately life-threatening or result in death or hospitalization but may jeopardize the subject or may require medical or surgical intervention to prevent one of the other outcomes listed in the above definition. These should also be considered serious. Examples of such events are invasive or malignant cancers, intensive treatment in an emergency room or at home for allergic bronchospasm, blood dyscrasias or convulsions that do not result in hospitalization, or development of drug dependency or drug abuse.

Additionally, the following protocol specific events will be reported as SAEs:

g. Haemoglobin values: a drop of ≥40% of baseline haemoglobin and / or all blood transfusions, and/or all haemoglobin values of < 5g/dL

h. Methaemoglobin values: all values of ≥20%, and /or values of ≥10% but <20% with associated clinical symptoms of methaemoglobinaemia.

10.2.1. Clinical Laboratory Abnormalities and Other Abnormal Assessments as AEs and SAEs

Abnormal laboratory findings (e.g., clinical chemistry, hematology, urinalysis) or other abnormal assessments (e.g., vital signs) that are judged by the investigator as **clinically significant** will be recorded as AEs or SAEs if they meet the definition of an AE or SAE. Clinically significant abnormal laboratory findings or other abnormal assessments that are detected during the study or are present at baseline and significantly worsen following the start of the study will be reported as AEs or SAEs. However, clinically significant abnormal laboratory findings or other abnormal assessments that are associated with the disease being studied, unless judged by the investigator as more severe than expected for the subject’s condition, or that are present or detected at the start of the study and do not worsen, will **not** be reported as AEs or SAEs.

The investigator will exercise his or her medical and scientific judgment in deciding whether an abnormal laboratory finding or other abnormal assessment is clinically significant.

10.3. Time Period, and Frequency of Detecting AEs and SAEs

From the time a subject consents to participate in the study until he or she has completed the study (including any follow-up period), all SAEs assessed as related to study participation (e.g., protocol-mandated procedures, invasive tests, or change in existing therapy) or related to a GSK concomitant medication, will be reported promptly to GSK.

10.4. Prompt Reporting of SAEs to GSK

SAEs will be reported promptly to GSK as described in the following table once the investigator determines that the event meets the protocol definition of an SAE.

10.4.1. Timeframes for Submitting SAE Reports to GSK

	Initial SAE Reports		Follow-up Information on a Previously Reported SAE	
Type of SAE	Time Frame	Documents	Time Frame	Documents
All SAEs	24 hrs	"SAE" data collection tool	24 hrs	Updated "SAE" data collection tool

10.5. AE and SAE Documentation and Follow-up Procedures

The investigator will review and adhere to the following procedures, which are outlined in detail in the AE/SAE section of the SRM:

- Method of Detecting AEs and SAEs
- Recording of AEs and SAEs
- Evaluating of AEs and SAEs
- Completion and Transmission of SAE Reports to GSK
- Follow-up of AEs and SAEs
- Post-study AEs and SAEs
- Regulatory Reporting Requirements for SAEs.

11. DATA ANALYSIS AND STATISTICAL CONSIDERATIONS

11.1. Hypotheses

The null hypothesis for the primary endpoint is that the parasitological cure rate at Day 28 (PCR corrected) in subjects treated with CDA, is clinically inferior to that for artemether-lumefantrine. The one-sided alternative hypothesis is that the parasitological cure rate for CDA subjects is non-inferior to that of artemether-lumefantrine.

Assessment of these hypotheses will be based on the lower bound of the two-sided 95% confidence interval for the treatment difference (CDA minus artemether-lumefantrine). It will be concluded that CDA is non-inferior if the lower bound is greater than -7%.

11.2. Study Design Considerations

11.2.1. Choice of Non-Inferiority Margin

The choice of a 7% non-inferiority margin is difficult to justify in a formal statistical manner as no placebo-controlled studies have ever been conducted for anti-malarial compounds for ethical reasons. Therefore, it is not possible to generate a meaningful indirect confidence interval for CDA – placebo in order to justify the choice of margin in terms of demonstration of efficacy relative to placebo. Consequently, a standard precedent for NI margin in malaria has not been adopted at this time. However, the following reasoning is useful in establishing the relevance of a 7% margin with respect to providing assurance that CDA has a clinically relevant superiority over placebo:

Effective therapies for malaria generally have observed efficacy rates of 90% or above, with the active comparator (Coartem), which is the current gold-standard, achieving at least 95%. Although there are no placebo-controlled data for any anti-malarial compound, the ‘untreated’ parasitological cure rate for this patient population would be 0% - some immune or semi-immune patients may recover from clinical symptoms but it is highly unlikely that any patients would have complete parasite clearance. Many individuals in malaria-endemic areas harbour malaria parasites but are asymptomatic [Chandler, 2006; Okocha, 2005; Njama-Meya, 2004; Anorlu, 2001].

Therefore, anti-malarial treatments achieve a very large and consistent treatment effect relative to ‘no treatment’. Additionally, the primary endpoint under consideration is highly objective. For these reasons, there is a large degree of confidence that if a 7% NI margin is used and CDA demonstrates non-inferiority to Coartem within the constraints of this margin, then CDA would have been shown to be statistically and clinically superior to placebo if a placebo-controlled trial had been performed.

With regard to establishing the clinical relevance of a 7% margin for showing there is no important loss of efficacy if CDA is used to treat this patient population instead of other licenced treatments, several different combination treatments are currently used interchangeably for P. fulciparum malaria. These are considered to achieve at least 90% PCR-corrected parasitological cure at 28-days. However, at the time of protocol development for this study (2005), there were very limited 28-day parasitological cure data available to assess the range of effects observed for the current treatments. Consequently, the assumption that Coartem achieves 95% cure was thought to be relatively conservative and therefore a 5% margin was considered to be too stringent as the true range of effects for the commonly-used anti-malarials could potentially be greater than this. Therefore, 7% was selected to allow some flexibility with respect to the true range of treatment effects across the various anti-malarial combinations.

However, more information regarding 28-day parasitological cure rates for current combination therapies is now available and this has been reviewed in order to re-assess the validity of a 7% NI margin:

- Pooled analysis of 11 randomised artemether-lumefantrine (Coartem) studies [Mueller, 2006]: PCR-corrected rate (Per Protocol population) = 97%
- Open-label study of artesunate-amodiaquine [Oyakhirome, 2007]: PCR-corrected rate (PP population) = 86%
- Randomised open-label study of artesunate-SP, artesunate-amodiaquine and artemether-lumefantrine (Coartem) [van den Broek, 2006]: PCR-corrected rates (PP population) =
    - 90% (artesunate-SP)
    - 98.5% (artesunate-amodiaquine)
    - 100% (artemether-lumefantrine)
- Open-label randomised study of artesunate-SP and artesunate-amodiaquine, [Hamour, 2005]: PCR-corrected rates (PP population) =
    - 91% (artesunate-SP)
    - 93% (artesunate-amodiaquine)

The published 28-day parasitological cure rates range from 86% to 100%, although differences in study design, methodology and analysis could account for this wide range of observed effects. However, within the Van den Broek study of 298 patients, the effects of three different anti-malarial combinations ranged from 90% to 100%. **Therefore, the choice of a 7% margin is still considered to be appropriate for this study.**

11.2.2. Sample Size Assumptions

A total of 650 evaluable patients in the CDA group and 325 evaluable patients in the artemether-lumefantrine group will provide **at least** 90% power to show non-inferiority of CDA to artemether-lumefantrine in terms of a one-sided hypothesis test with a 2.5% significance level for the primary endpoint of the parasitological cure rate at Day 28 (PCR corrected). This is based on using a 7% non-inferiority margin and a 2:1 allocation ratio, assuming 93% efficacy for CDA and 95% efficacy for artemether-lumefantrine amongst patients evaluable for the primary per-protocol population.

A 2:1 ratio has been chosen to preferentially collect information on the combination of CDA to artemether-lumefantrine, due to an interest in collecting additional safety data on the CDA arm.

In order to accommodate up to 30% loss of evaluable data due to missing data where patients are lost to follow-up, or have been lost to analysis due to retreatment for a new infection, it is planned that a total of 1395 patients will be enrolled (930 to CDA, 465 to artemether-lumefantrine).

At the time of sample size calculation for this study, the Day 28 PCR corrected Parasitological Cure Rate of artemether-lumefantrine was estimated to be 95% with values in the literature ranging between 93.5% to 97.5%. These parasitological cure rates have been observed in different trials run in different regions of the world at different times and in different populations.

It is expected that the efficacy rate for CDA will similarly be 95% (ie. a 0% treatment difference). However, due to the lack of data for CDA, a more conservative treatment difference is being used to calculate the sample size with 95% for artemether-lumefantrine and 93% for CDA (ie. a 2% treatment difference).

The impact on the power of the study due to deviations from assumed response rates is discussed in Section 11.2.3.

A single primary endpoint analysis has been defined for this study with a single treatment comparison. No adjustments for multiplicity are therefore required.

11.2.3. Sample Size Sensitivity

The robustness and sensitivity of the above sample size should be considered in order to assess the impact of different circumstances on the power of the per-protocol analysis for the primary endpoint.

Power will vary if the observed parasitological cure rate at day 28 for artemether-lumefantrine is different to the expected 95%. As stated in Section 11.2.1, published values from trials run in different regions of the world at different times and in different populations, range from 93.5% to 97.5%. The data presented shows for different scenarios how the power of the analysis decreases if the parasitological cure rate at day 28 increases above 95%, within this observed range:

Table 1 Variation in power with different artemether-lumefantrine parasitological cure rates

Observed CDA rate: 93.1% (605/650)	Artemether-lumefantrine rate			
	95.1% (309/325)	95.7% (311/325)	96.3% (313/325)	96.9% (315/325)
Power[1]	90.6%	84.3%	75.4%	63.2%

1. Given a one-sided hypothesis at 2.5% significance level, a 7% non-inferiority margin, 650 evaluable CDA subjects and 325 evaluable artemether-lumefantrine subjects

Also as stated in Section 11.2.1, although it is expected that the efficacy rate for CDA will also be 95%, due a lack of available data for day 28, a conservative rate of 93% has been assumed. The data presented shows for different scenarios how the power of the analysis varies if the rate for CDA varies between 92% and 95%:

Table 2 Variation in power with different CDA parasitological cure rates

Observed artemether-lumefantrine rate: 95.1% (309/325)	CDA rate			
	92.0% (598/650)	92.5% (601/650)	93.5% (608/650)	94.0% (611/650)
Power	70.8%	80.8%	95.1%	97.7%

The impact on the power of the per-protocol analysis of observing a higher drop out rate than the assumed 30% of subjects enrolled, is considered below. This would result in there being fewer than the 650 CDA and 325 evaluable artemether-lumefantrine subjects available for the analysis to have 90% power, even if other sample-size assumptions are met:

Table 3 Impact of a higher drop out rate on the power of the study

	CDA	Artemether-lumefantrine	Power
Number of subjects enrolled:	930	465	
No. evaluable (% dropouts)	605 (34.9%)	305 (35.1%)	
Parasitological cure rate at Day 28	93.1% (563/605)	95.1% (290/305)	88.3%

Thus there is only a small loss of power for the primary analysis if a 35% withdrawal/non-evaluability rate is observed (rather than 30%) but other assumptions for CDA and artemether-lumefantrine are met.

11.2.4. Sample Size Re-estimation

No sample size re-estimation is planned in this study.

11.3. Data Analysis Considerations

11.3.1. Analysis Populations

The following populations will be evaluated:

- Intent to treat
- Per Protocol
- Pharmacokinetic population

Intent-to-treat population (ITT)

All subjects who were randomised and have received any dose of the study medication, irrespective of whether they vomited, will be included in the intent-to-treat population. The intent-to-treat analysis of efficacy will be a supportive analysis population for the primary efficacy endpoint and will also be used for all secondary efficacy and safety analyses.

Per-protocol populations (PP)

The Per Protocol (PP) population will consist of all subjects in the ITT population not identified as major protocol violators. The major protocol violations for which patients would be excluded from the PP population will be described in the Reporting and Analysis Plan and will represent violations that may impact the efficacy endpoints for

which non-inferiority comparisons are being made. The decision to exclude a subject from the PP population will be made prior to breaking the blind. This population will be used for primary analysis of the day 28 cure rate and supportive analysis of the secondary efficacy endpoints,

11.3.2. Analysis Data Sets

Data sets will contain a flag to identify for which analysis populations subjects are eligible. Additionally, modified population datasets will be created for the purposes of sensitivity analyses. Full details of these analysis datasets will be given in the Reporting Analysis Plan.

11.3.3. Treatment Comparisons

11.3.3.1. Primary Comparisons of Interest

The primary comparison of interest will be in the parasitological cure rate at Day 28 (PCR corrected) for artemether-lumefantrine versus CDA using the Day 28 Per Protocol Population.

The comparison will be made for CDA minus artemether-lumefantrine and a two-sided 95% confidence interval for the treatment difference will be used, giving a nominal significance level for falsely rejecting the null hypothesis of 2.5%. As only one comparison is being studied, no adjustment for multiple comparisons is required. Non-inferiority will be concluded if the lower bound of the two-sided 95% confidence interval is no less than than -7%.

11.3.3.2. Other Comparisons of Interest

A key supportive analysis using the Intent-to-Treat population will also be conducted as well as other sensitivity analyses to confirm consistency of the primary analysis results under varied assumptions or methods. (No adjustments are made for these sensitivity analyses).

Comparisons of CDA and artemether-lumefantrine may be provided for other secondary endpoints for descriptive purposes.

11.3.4. Interim Analysis

Interim analyses of efficacy and safety data are planned as follows:

1. An Independent Data Monitoring Committee (IDMC) is planned to review the risk:benefit ratio of CDA, and to achieve this will monitor both efficacy and safety aspects of this study. The details of the frequency and type of analyses will be described in the IDMC Charter.
2. An internal Safety Review Team (SRT) is planned to review the safety data on an ongoing basis. These reviews will be conducted while blinded to treatment details.

11.3.5. Key Elements of Analysis Plan

11.3.5.1. Data derivation

For the primary endpoint, Parasitological Cure Rate at Day 28, in order to be classified as 'cured' at Day 28, subjects must demonstrate parasite clearance of their initial infection either at day 7 (+/- 1 day) if tested at this time point, or earlier (during day 3 to 6) if not tested at day 7, and must remain clear of their initial infection at day 28 (- 1, +3 days) following PCR-correction. These are the minimum data points required for a subject to be included in the primary analysis.

If a positive parasite count is detected at any intermittent timepoint day 7 to day 28, the patient will be programmatically classified as a 'failure'.

However, PCR analysis will be conducted on samples with positive counts at any intermittent timepoint day 7 to day 28 to assess if the parasites represent a recrudescence (re-infestation of the original baseline parasite infection) or a re-infection (infection with a new parasite after the baseline assessment). If a positive parasite count is determined to be a re-infection then the data for this subject will not be included as a 'failure' but rather will be excluded from the analysis since a new infection does not demonstrate a failure to treat the original infection.

Similarly, for the Day 14 analyses, patients must have data at either day 7 (+/-1 day) if tested at this timepoint or earlier (day 3 to 6) if not tested at day 7, and must remain clear of their initial infection at day 14 (-1,+3days) following PCR-correction. These are the minimum data points required for a subject to be classified as a responder in the Day 14 analyses. The Day 42 analysis would require the same timepoints; however, instead of Day 14 (-1,+3 days), Day 42 (-1,+3 days) would be required.

11.3.5.2. Missing data

For the ITT population, subjects will not be excluded from any statistical analyses with the exception of efficacy analyses for PCR-confirmed cases of new malarial infection. For the primary endpoint for this population, an imputation of treatment failure will be made for subjects without a day 28 parasitological assessment. However, all data summaries of the ITT population will be performed with no replacement of missing CRF efficacy and safety data. Patients with missing data at the relevant timepoint will be excluded from analyses for the Per Protocol population eg patients withdrawing from the study prior to day 28 will be excluded from the primary endpoint analysis for the Per Protocol population. Full details of how missing CRF data will be handled will be described in the Reporting Analysis Plan.

11.3.5.3. Assessment windows

As described in Section 11.3.5.1, the following assessment windows will be applied in order to be considered evaluable for the primary analysis, subjects must have at least one blood slide recorded during day 3 to day 5 or at day 7 (+/-1 day) and at day 28 (-1, +3 days).

Similarly, for the day 14 analyses, the assessment window will be day 14 (-1, +3 days); and for the day 42 analyses, the assessment window will be day 42 (-1, +3 days).

11.3.5.4. Methods of Analysis

For the primary analysis, 95% C.I.'s will be unadjusted for covariates.

11.3.5.5. Subgroup analyses

No formal subgroup analyses are planned. However for investigative purposes 95% confidence intervals may be calculated within pooled centres, which will be pre-specified in the RAP. Summaries of the primary endpoint based on Age, Gender, and Ethnicity (if appropriate) will also be provided.

11.3.5.6. Deviations from the Planned Analyses

Any deviations from the analyses planned as part of this protocol will be documented in the Reporting and Analysis Plan (RAP), prior to unblinding.

11.3.5.7. Efficacy Analyses

The primary analysis is the Per Protocol analysis of the Parasitolgical Cure Rate (PCR-corrected) at Day 28.

A key supportive analysis will be the Intent-to-Treat analysis of the Parasitological Cure Rate (PCR-corrected) at Day 28, with missing data imputed as treatment failure

Additional sensitivity analyses of the primary endpoint will be performed to assess the robustness of the primary analysis to modifications in assumptions and methods. Analyses such as the following will be performed:

- Intent-to-Treat analysis of the Parasitological Cure Rate (PCR-corrected) at Day 28, with missing data excluded.
- Per Protocol analysis of the Parasitological Cure Rate (without PCR correction) at Day 28.

Secondary Analyses will include:

- Parasitological cure rate of CDA vs artemether-lumefantrine at day 14 (PCR corrected).
- Parasitological cure rate of CDA vs artemether-lumefantrine at day 42 (PCR corrected).
- Adequate Clinical and Parasitological Response rate (ACPR [World Health Organisation, 2003]) at day 14, 28 and 42 (all PCR corrected).
- Asexual parasite densities over time for CDA vs. artemether-lumefantrine.
- PCT of CDA vs artemether-lumefantrine.

- Gametocyte density over time of CDA vs artemether-lumefantrine.
- FCT of CDA vs artemether-lumefantrine.

Details of theses analyses will be documented in the Reporting and Analysis Plan (RAP).

11.3.5.8. Safety Analyses

All safety reporting will be based on the intent-to-treat population.

11.3.5.8.1. Extent of Exposure

Subjects will be dosed on Day 0, 1 and 2 of the study. The extent of exposure will be the number of doses of study medication administered to the subject (regardless of whether vomited).

The duration of exposure to study medication will be defined as date of last dose of active study medication – date of first dose of study medication + 1. Extent and duration of exposure will be summarised using a frequency distribution for number of doses and number of days.

11.3.5.8.2. Adverse Events

Adverse Event (AE) reporting will be performed using the MedDRA (Medical Dictionaries for Regulatory Activities) coding system. Each AE coded using the MedDRA system can be associated with more than one system organ class (SOC). However, for reporting purposes, an AE will be associated with the primary system organ class only.

Counting of adverse events will be based on the number of subjects – not the number of AEs. For example, if a subject reports the same AE on three occasions within a time interval, that AE will only be counted once. Subjects reporting more than one AE in a system organ class will only be counted once in the system organ class total.

All treatment-emergent AE's (i.e. with onset time on or after that of first dose), will be listed and a summary table will be prepared showing the incidence of all recorded AEs by treatment group. However, all other AE tabulations will be produced excluding occurrences of PCR-confirmed P. fulciparum recrudescence. Such events will be classified as treatment failures for the purposes of efficacy analyses but will not be considered AEs for the purposes of comparing the AE profiles between the two treatment groups. Occurrences of new malarial infection, as confirmed by PCR will remain as adverse events and will be reported in all the AE outputs. The following summary tables will be produced for all AEs except those confirmed as recrudescences:

AE's by preferred term and SOC, AE's in descending order of frequency, AE's by maximum severity (mild, moderate or severe).

AE's considered by the investigator to have a reasonable possibility of being related to treatment ('drug-related' AEs) will be summarised by preferred term and SOC.

AE's leading to premature withdrawal from treatment will be summarised by preferred term and SOC.

AE's leading to premature withdrawal from study will be summarised by preferred term and SOC.

AE's that are considered to be haematologically-related will be identified for reporting by clinical review of blinded AE data. Haematological AE's will be summarised by preferred term and SOC.

Serious adverse events will be summarised by preferred term and SOC.

11.3.5.8.3. Clinical Laboratory Evaluations

Clinical laboratory data (clinical chemistry and haematology) will be summarised by the mean, median standard deviation, minimum and maximum values by treatment group and time point.

Laboratory data will also be evaluated by tabulating the number and percentage of subjects in each treatment group with values outside specified threshold values of clinical concern. (These may include values outside of the normal range, outer range of clinical concern, and other values of clinical concern.) These safety analyses will be defined in the analysis plan as appropriate.

11.3.5.8.4. G6PD Evaluations

The proportion of subjects with G6PD deficiency will be reported. Clinical laboratory data will be summarised by treatment group and G6PD status.

11.3.5.9. Pharmacokinetic Analyses

Plasma concentration-time data for DDS, CPG, CCG, ART and DHA will be tabulated for each subject receiving CDA. Population pharmacokinetic methods will be performed using software such as NONMEM or other currently acceptable methods to assess the pharmacokinetics of CDA components, if data are appropriate. To support the population PK analysis, the data from this study may be combined with data from other studies. Mean population PK parameters will be assessed taking into account demographic variables (such as weight, age, gender), and concomitant medications. If data permit, the relationship between CDA component concentrations and selected adverse events will be explored.

12. STUDY CONDUCT CONSIDERATIONS

12.1. Regulatory and Ethical Considerations, Including the Informed Consent Process

GSK will obtain favorable opinion/approval to conduct the study from the appropriate regulatory agency in accordance with any applicable country-specific regulatory requirements prior to a site initiating the study in that country.

The study will be conducted in accordance with all applicable regulatory requirements.

The study will also be conducted in accordance with "good clinical practice" (GCP), all applicable subject privacy requirements, and, the guiding principles of the Declaration of Helsinki. This includes, but is not limited to, the following:

- IEC/IRB review and favorable opinion/approval to conduct the study and of any subsequent relevant amended documents
- Subject informed consent
- Investigator reporting requirements

GSK will provide full details of the above either verbally, in writing or both.

Written informed consent will be obtained for each subject before he or she can participate in the study.

12.2. Quality Control (Study Monitoring)

In accordance with applicable regulations, GCP, and GSK procedures, GSK monitors will contact the site prior to the start of the study to review with the site staff the protocol, study requirements, and their responsibilities to satisfy regulatory, ethical, and GSK requirements. When reviewing data collection procedures, the discussion will also include identification, agreement and documentation of data items for which the eCRF will serve as the source document.

GSK will monitor the study consistent with the demands of the study and site activity to verify that the:

- Data are authentic, accurate, and complete.
- Safety and rights of subjects are being protected.
- Study is conducted in accordance with the currently approved protocol and any other study agreements, GCP, and all applicable regulatory requirements.

The investigator and the head of the medical institution (where applicable) agrees to allow the monitor direct access to all relevant documents.

12.3. Quality Assurance

To ensure compliance with GCP and all applicable regulatory requirements, GSK may conduct a quality assurance audit. Regulatory agencies may also conduct a regulatory inspection of this study. Such audits/inspections can occur at any time during or after completion of the study. If an audit or inspection occurs, the investigator and institution agree to allow the auditor/inspector direct access to all relevant documents and to allocate his/her time and the time of his/her staff to the auditor/inspector to discuss findings and any relevant issues.

12.4. Study and Site Closure

Upon completion or premature discontinuation of the study, the monitor will conduct site closure activities with the investigator or site staff, as appropriate, in accordance with applicable regulations, GCP, and GSK procedures.

In addition, GSK reserves the right to temporarily suspend or prematurely discontinue this study at any time for reasons including, but not limited to, safety or ethical issues or severe non-compliance. For multicenter studies, this can occur at one or more or at all sites. If GSK determines such action is needed, GSK will discuss this with the investigator or the head of the medical institution (where applicable), including the reasons for taking such action, at that time. When feasible, GSK will provide advance notification to the investigator or the head of the medical institution, where applicable, of the impending action prior to it taking effect.

GSK will promptly inform all other investigators or the head of the medical institution (where applicable), and/or institutions conducting the study if the study is suspended or terminated for safety reasons. GSK will also promptly inform the regulatory authorities of the suspension or termination of the study and the reason(s) for the action. If required by applicable regulations, the investigator or the head of the medical institution (where applicable) must inform the IEC/IRB promptly and provide the reason for the suspension or termination.

12.5. Records Retention

Following closure of the study, the investigator or the head of the medical institution (where applicable) must maintain all site study records, except for those required by local regulations to be maintained by someone else, in a safe and secure location. The records must be maintained to allow easy and timely retrieval, when needed (e.g., audit or inspection), and, whenever feasible, to allow any subsequent review of data in conjunction with assessment of the facility, supporting systems, and staff. Where permitted by local laws/regulations or institutional policy, some or all of these records can be maintained in a format other than hard copy (e.g., microfiche, scanned, electronic); however, caution needs to be exercised before such action is taken. The investigator must assure that all reproductions are legible and are a true and accurate copy of the original, and meet accessibility and retrieval standards, including re-generating a hard copy, if required. Furthermore, the investigator must ensure there is an acceptable

back-up of these reproductions and that an acceptable quality control process exists for making these reproductions.

GSK will inform the investigator of the time period for retaining these records to comply with all applicable regulatory requirements. The minimum retention time will meet the strictest standard applicable to that site for the study, as dictated by any institutional requirements or local laws or regulations, or GSK standards/procedures; otherwise, the retention period will default to 15 years.

The investigator must notify GSK of any changes in the archival arrangements, including, but not limited to, the following: archival at an off-site facility, transfer of ownership of the records in the event the investigator leaves the site.

12.6. Provision of Study Results and Information to Investigators

When required by applicable regulations, the investigator signatory for the clinical study report will be determined at the time the report is written. When the clinical study report is completed, GSK will provide the investigator with a full summary of the study results. In addition, the investigator will be given reasonable access to review the relevant statistical tables, figures, and reports and will be able to review the results for the entire study at a GSK site or other mutually agreeable location.

Information on the progress of the study will be provided at intervals though newsletters and verbal presentations, so that all those involved in the study including study subjects can be kept fully informed. On completion of the study, in addition to the publication of data in the scientific literature, information on the outcome will be provided to subjects and to their communities in an appropriate manner (for example through the local media, through workshops or by direct contact with the subjects). Policy makers will be informed through the normal scientific channels, and local regulatory authorities will be provided with a summary of the outcomes of the study.

GSK will provide the investigator with the randomization codes for their site after the statistical analysis for the entire study has been completed.

12.7. Data Management

The data collection tool for this study will be GSK-defined electronic case report forms (eCRFs). In all cases, subject initials will not be collected nor transmitted to GSK. Subject data necessary for analysis and reporting will be entered/transmitted into a validated database or data system. Clinical data management will be performed in accordance with applicable GSK standards and data cleaning procedures.

12.8. Independent Data Monitoring Committee (IDMC)

An IDMC will be utilized during the conduct of this study. An IDMC is generally assembled when there are significant safety or efficacy issues that warrant external objective medical and/or statistical review in order to protect the ethical and safety interests of subjects and to protect the scientific validity of the study. The schedule of any planned interim analysis and the analysis plan for IDMC review is described in the charter. A copy of the IDMC charter is available from GSK upon request.

13. REFERENCES

Alloueche A, Bailey W, Barton S, Bwika J, Chimpeni P, Falade CO, Fehintola FA, Horton J, Jaffar S, Kanyok T, Kremsner PG, Kublin JG, Lang T, Missinou MA, Mkandala C, Oduola AM, Premji Z, Robertson L, Sowunmi A, Ward SA, Winstanley PA. Comparison of chlorproguanil-dapsone with sulfadoxine-pyrimethamine for the treatment of uncomplicated falciparum malaria in young African children: double-blind randomised controlled trial. *Lancet.* 2004; **363**: 1843-8.

Anonymous. Artemisinin. *Trans R Soc Trop Med Hyg*, 1994; **88**(Suppl. 2): 1-65.

Anorlu RI, Odum CU, Essien EE. Asymptomatic malaria parasitaemia in pregnant women at booking in a primary health care facility in a periurban community in Lagos, Nigeria. Afr J Med Med Sci. 2001;30 Suppl:39-41

Chandler CI, Drakeley CJ, Reyburn H, Carneiro I. The effect of altitude on parasite density case definitions for malaria in northeastern Tanzania. Trop Med Int Health. 2006 Aug;11(8):1178-84.

Chlorproguanil,dapsone,artesunate (CDA) Investigator’s Brochure GM2002/00293/01

Falade C, Makanga M, Premji Z, Ortmann C-E, Stockmeyer M and Ibarra de Palacios P. Efficacy and safety of artemether–lumefantrine (Coartem) tablets (six-dose regimen) in African infants and children with acute, uncomplicated falciparum malaria. *Trans R Soc Trop Med Hyg,* 2005; **99**(6): 459-67.

Hamour S, Melaku Y, Keus K, Wambugu J, Atkin S, Montgomery J, Ford N, Hook C, Checchi F. Malaria in the Nuba Mountains of Sudan: baseline genotypic resistance and efficacy of the artesunate plus sulfadoxine-pyrimethamine and artesunate plus amodiaquine combinations. Trans R Soc Trop Med Hyg. 2005 Jul;99(7):548-54.

Kublin JG, Dzinjalamala FK, Kamwendo DD, Malkin EM, Cortese JF, Martino LM, Mukadam RA, Rogerson SJ, Lescano AG, Molyneux ME, Winstanley PA, Chimpeni P, Taylor TE, Plowe CV. Molecular markers for failure of sulfadoxine-pyrimethamine and chlorproguanil-dapsone treatment of Plasmodium falciparum malaria. J Infect Dis. 2002 **185**:380-8.

Lefevre G, Looareesuwan S, Treeprasertsuk S, Krudsood S, Silachamroon U, Gathmann I, Mull R, Bakshi R. A clinical and pharmacokinetic trial of six doses of artemether-lumefantrine for multidrug-resistant *Plasmodium falciparum* malaria in Thailand. *Am J Trop Med Hyg*. 2001; **64**: 247-56.

Mueller EA, van Vugt M, Kirch W, Andriano K, Hunt P, de Palacios PI. Efficacy and safety of the six-dose regimen of artemether-lumefantrine for treatment of uncomplicated Plasmodium falciparum malaria in adolescents and adults: a pooled analysis of individual patient data from randomized clinical trials. Acta Trop. 2006 Nov;100(1-2):41-53. Epub 2006 Oct 12.

Mutabingwa T,Nzila A,Mberu E,Nduati E,Winstanley P,Hills E,Watkins W. Chlorproguanil-dapsone for treatment of drug-resistant falciparum malaria in Tanzania. *Lancet*, 2001; **358**: 1218-23.

Njama-Meya D, Kamya MR, Dorsey G. Asymptomatic parasitaemia as a risk factor for symptomatic malaria in a cohort of Ugandan children. Trop Med Int Health. 2004 Aug;9(8):862-8

Nzila MA, Mberu EK, Sulo J, Dayo H, Winstanley PA, Sibley CH, Watkins WM. Towards an understanding of the mechanism of pyrimethamine/sulfadoxine resistance in *Plasmodium falciparum*: the genotyping of dihydrofolate reductase and dihydropteroate synthase of Kenyan parasites. *Antimicrob Agent Chemother*, 2000a; **44**(4): 991-6.

Nzila AM, Nduati E, Mberu EK, Hopkins Sibley C, Monks SA, Winstanley PA, Watkins WM. Molecular evidence of greater selective pressure for drug resistance exerted by the long-acting antifolate Pyrimethamine/Sulfadoxine compared with the shorteracting Chlorproguanil/Dapsone on Kenyan *Plasmodium falciparum. J Infect Dis*, 2000b Jun; **181**(6): 2023-8.

Okocha EC, Ibeh CC, Ele PU, Ibeh NC. The prevalence of malaria parasitaemia in blood donors in a Nigerian teaching hospital. J Vector Borne Dis. 2005 Mar;42(1):21-4.

Oyakhirome S, Potschke M, Schwarz NG, Dornemann J, Laengin M, Salazar CO, Lell B, Kun JF, Kremsner PG, Grobusch MP. Artesunate--amodiaquine combination therapy for falciparum malaria in young Gabonese children. Malar J. 2007 Mar 12;6:29.

Snounou G, Beck H-P. The use of PCR genotyping in the assessment of recrudescence or reinfection after antimalarial drug treatment. Parasitol Today, 1998; 14: 462-7.

Watkins WM, Mberu EK, Winstanley PA and Plowe C. The efficacy of antifolate antimalarial combinations in Africa : a predictive model based on pharmacodynamic and pharmacokinetic analyses. *Parasitol Today*, 1997; **13**: 459-64.

van den Broek I, Kitz C, Al Attas S, Libama F, Balasegaram M, Guthmann JP. Efficacy of three artemisinin combination therapies for the treatment of uncomplicated Plasmodium falciparum malaria in the Republic of Congo. Malar J. 2006 Nov 24;5:113.

van Vugt M, Looareesuwan S, Wilairatana P, McGready R, Villegas L, Gathmann I, Mull R, Brockman A, White NJ, Nosten F. Artemether-lumefantrine for the treatment of multidrug-resistant falciparum malaria. *Trans R Soc Trop Med Hyg,* 2000; **94**: 545-8.

Vugt MV, Wilairatana P, Gemperli B, Gathmann I, Phaipun L, Brockman A, Luxemburger C, White NJ, Nosten F, Looareesuwan S. Efficacy of six doses of artemether-lumefantrine (benflumetol) in multidrug-resistant *Plasmodium falciparum* malaria. *Am J Trop Med Hyg,* 1999; **60**: 936-42.

Watkins WM, Mberu EK, Winstanley PA, Plowe CV. More on 'The efficacy of antifolate antimalarial combinations in Africa'. Parasitol Today, 1999; 15: 131-2

Wernsdorfer WH, Payne D. The dynamics of drug resistance in *Plasmodium falciparum*. *Pharm Ther*, 1991; **50**: 905-121.

White NJ, Nosten F, Looaresuwan S, Watkins WM, Marsh K, Snow RW, Kokwaro G, Ouma J, Hien TT, Molyneux M, Taylor T, Newbold CI, Ruebush TK, Danis M, Greenwood B, Anderson RM, Olliaro P. 'Averting a malaria disaster'. *Lancet*, 1999a; **353**: 1965-7.

White N, Nosten F, Hien TT, Watkins W, Olliaro P. 'Averting a malaria disaster'. *Lancet*, 1999b; **354**: 1389-90.

White NJ, Olliaro PL. Strategies for the prevention of antimalarial drug resistance: rationale for combination chemotherapy for malaria. *Parasitol Today*, 1996; **12**: 399-401.

White NJ. The assessment of antimalarial drug efficacy. *Trends Parasitol*, 2002; **18**: 458-64.

World Health Organisation. Severe falciparum malaria. *Trans R Soc Trop Med Hyg,* 2000; **94**, supplement 1.

World Health Organisation. Antimalarial Drug Combination Therapy. WHO 2001, Report of a WHO Technical Consultation.

World Health Organisation. Report of an RBM/TDR informal consultation of clinical investigators on the safety and efficacy with artemisinin compunds in pregnancy. WHO 2002. July 22-23, Geneva.

World Health Organisation. Assessment and monitoring of antimalarial drug efficacy for the treatment of uncomplicated falciparum malaria. WHO 2003.

WHO Essential Medicines Library – EMLib. WHO Model Formulary English edition 2004: http://mednet3.who.int/EMLib/wmf/English/WMFwholecorrected.doc. Last access: 24 June 2005.

Appendices

Appendix 1: Time and Events Table

Changes have been made to the time & events table to reflect the modification of the assessment times, however the table has not been reproduced here to conserve space.

See next page

Assessment day	Day 0			Day 1	Day 2	Day 3	Day 4	Day 5	Day 6	Day 7	Day 14	Day 28	Day 42	Extra visit
Time (hr)	screening	Pre-dose	0	24	48	72								
Procedure														
Obtain informed consent from parent / guardian and assent from child.	X													
Weigh subject, record length or height, collect demography data	X													
Clinical assessment	X	X	X	X	X	X				X	X	X	X	X
Record medical history	X													
Tympanic temperature measured	X	X	X (every 8hrs)	X [1] (every 8hrs)	X [1] (every 8hrs)	X				X	X	X	X	X
Urine test for CQ	X													
Thumbprick - blood slide for parasite count (10ul)	X	X	X (every 8hrs)	X [1] (every 8hrs)	X [1] (every 8hrs)	X				X	X	X	X	X
Thumbprick - blood sample to filter paper for parasite PCR (2 drops)		X								X	X	X	X	X
Venous blood sample for haematology (2ml)	X			X [2]	X [2]	X				X	X	X	X	(X) [3]
Venus blood sample for clinical chemistry (2ml)		X				X				X	(X) [4]	(X) [4]	X	(X) [3]
Serum pregnancy test [5] (4 drops)	X													
Urine pregnancy test [5] (4 drops)													X	
Blood sample for human G6PD PCR (10ul)		X												
G6PD phenotype test (aliquot from haematology sample)		X										(X) [6]		
Population PK sampling (2ml)			X [7]	X [7]	X [7]					X [7]				
Study drug administration (twice daily dosing)			X [8]	X	X									
Field worker home visit							X	X	X					
Concomitant medication recorded	X	X	X	X	X	X				X	X	X	X	X
AE and SAE reporting	X	X	X	X	X	X				X	X	X	X	X
Rescue medication if treatment failure			X	X	X	X				X	X	X	X	X

1. Including pre-dose assessments on days 1 and 2
2. Day 1 and 2 assessment should be before the first dose each day
3. At clinician's discretion
4. Samples should only be taken on day 14 or 28 if the previous results were abnormal.
5. Female subjects of child-bearing potential. Serum day 0, urine day 42 or on early withdrawal
6. Repeat phenotype test if drop in haemoglobin of 40% or greater from baseline (screening) once Hb has recovered, or if day 0 retic levels were high
7. Up to five blood samples for population PK analysis will be collected from each subject. These samples will be collected within varying time windows: 0.25-0.5h, 1-3h, 4-8h, 12-24h and day 7
8. Second dose day 0 should be ~8hrs after first dose.

Appendix 2: Country Specific Requirements

None.

Appendix 3: Dosing of study drugs

CDA

CDA tablets will be made in two sizes, to facilitate dosing of small children and infants:

CDA 12:15:24 tablets will contain 12mg CPG, 15mg DDS and 24mg artesunate.

CDA 60:75:120 tablets will contain 60mg CPG, 75 mg DDS and 120mg artesunate.

3-dose course: The following number of tablets should be taken at initial diagnosis after randomization, then once daily for the following two days.

12:15:24 CDA tablets								
No. of tablets	min weight	max weight	max mg/kg CPG	min mg/kg CPG	max mg/kg DDS	min mg/kg DDS	max mg/kg ART	min mg/kg ART
1.0	5.00	<7	2.40	1.74	3.00	2.17	4.80	3.48
1.5	7.00	<10	2.57	1.82	3.21	2.27	5.14	3.64
2.0	10.00	<13	2.40	1.86	3.00	2.33	4.80	3.72
60:75:120 CDA tablets								
No. of tablets	min weight	max weight	max mg/kg CPG	min mg/kg CPG	max mg/kg DDS	min mg/kg DDS	max mg/kg ART	min mg/kg ART
0.5	13.00	<19	2.31	1.59	2.88	1.98	4.62	3.17
1.0	19.00	<31	3.16	1.94	3.95	2.43	6.32	3.88
1.5	31.00	<46	2.90	1.96	3.63	2.45	5.81	3.92
2.0	46.00	-	2.61		3.26	-	5.22	-

Artemether-lumefantrine (Coartem)

Each tablet contains 20mg artemether and 120mg lumefantrine.

6-dose course: The following number of tablets should be taken at initial diagnosis after randomisation, then 8 hours after initial dose, and twice daily for the following two days.

- 5 - <15kg 1 tablet
- 15 - <25kg 2 tablets
- 25 - <35kg 3 tablets
- ≥35kg 4 tablets

Appendix 4: Investigators and collaborators associated with Kintampo Health Research Centre, Ghana

Kintampo Health Research Centre Team:

Seth Owusu-Agyei, MSc, PhD, Kintampo Health Research Centre, Ghana Health Service, P. O. Box 200, Kintampo Brong Ahafo, Ghana

Kwaku Poku Asante, MD, MPH, Kintampo Health Research Centre, Ghana Health Service, P. O. Box 200, Kintampo Brong Ahafo, Ghana

Ruth Owusu, MD, MPH, Kintampo Health Research Centre, Ghana Health Service, P. O. Box 200, Kintampo Brong Ahafo, Ghana

John Gyapong, MD, PhD, Kintampo Health Research Unit, Ghana Health Service, Accra, Ghana

London School of Hygiene and Tropical Medicine Team:

Daniel Chandramohan, MD MSc, London School of Hygiene and Tropical Medicine, UK.

Brian Greenwood, MD, MB BChir, BA, London School of Hygiene and Tropical Medicine, UK.

Appendix 5: Protocol Amendment 1 changes

Changes in Protocol amendment No.1.

This protocol amendment applies to Ghana only.

At the request of the IEC for the Kintampo site, all investigators and their collaborators need to be listed in the protocol.

The following investigators and collaborators will therefore be captured in Appendix 4.

Kintampo Health Research Centre Team:

Seth Owusu-Agyei, MSc, PhD, Kintampo Health Research Centre, Ghana Health Service, P. O. Box 200, Kintampo Brong Ahafo, Ghana

Kwaku Poku Asante, MD, MPH, Kintampo Health Research Centre, Ghana Health Service, P. O. Box 200, Kintampo Brong Ahafo, Ghana

Ruth Owusu, MD, MPH, Kintampo Health Research Centre, Ghana Health Service, P. O. Box 200, Kintampo Brong Ahafo, Ghana

John Gyapong, MD, PhD, Health Research Unit, Ghana Health Service, Accra, Ghana

London School of Hygiene and Tropical Medicine Team:

Daniel Chandramohan, MD MSc, London School of Hygiene and Tropical Medicine, UK.

Brian Greenwood, MD, MB BChir, BA,London School of Hygiene and Tropical Medicine, UK.

Appendix 6: Protocol Amendment 2 changes

Rationale for changes:

1. Change in age for inclusion.

The lower limit for inclusion into the current study has been increased from ≥3months to ≥12months. This is in light of recent juvenile toxicology data, in which the oral administration of CDA and CCG to 4 day old rat pups was progressively lethal at doses similar to those to be used in this phase III study. This was an unexpected finding, as adult rats had tolerated this combination in previous studies. However, we have previously dosed children ≥12months in the dose-ranging phase II study, and a thorough evaluation of the child data by age has not identified any trend in laboratory data or adverse events with age.

Therefore it is the recommendation of GSK and the CDA development partners to increase the lower age limit for inclusion into this study to ≥12months, corresponding to a weight of ≥7.5kg, until further data are available. A subsequent protocol amendment may look to reduce the age limit back to ≥3 months, once additional juvenile toxicology studies have been completed.

Changes to the protocol are as follows:

Protocol Summary, Study Population

Nine hundred male and female subjects presenting with acute uncomplicated *Plasmodium falciparum* malaria will be recruited. Eligible subjects will be aged *≥12months*, and weigh *≥7.5kg*.

5.2. Eligibility Criteria, Inclusion Criteria

A subject will be eligible for inclusion in this study only if all of the following criteria apply:

4. Age *≥12months*, no upper age limit.

5. Weight *≥7.5kg*, no upper weight limit

2. Addition of screening for sulphonamide drugs in all subjects.

A G6PD deficient patient (GdA-) who suffered haemolysis after malaria treatment in an on-going LAPDAP study had been treated with sulphadoxine/pyrimethamine (S/P) ~2 weeks before enrolment. We therefore propose to test for sulphonamide drugs in the urine of all subjects at enrolment. This screening for sulphonamide drugs will help us to evaluate the potential risk associated with a previous treatment of S/P or another sulphonamide drugs in our study subjects, and in particular in G6PD deficient subjects, who may present with haemolysis after treatment with CDA (dapsone).

Changes to the protocol are as follows:

6.1. Demographic and Baseline Assessments

- Urine test for sulphonamide drugs and chloroquine, *all subjects* (see Study Reference Manual)

6.4. Urine testing for antimalarial drugs

A urine sample will be taken for *all subjects to test for the presence of sulphonamide drugs at screening, as part of the assessment for eligibility. The same urine sample should be used to test for the presence of chloroquine*.

3. Clarification of previous malaria episodes

Further to discussions with the study investigators, it was agreed that the age for reporting previous malaria episodes should be increased to children aged ≤ 2years.

Changes to the protocol are as follows:

6.1. Demographic and Baseline Assessments

- Data on previous malaria episodes *in children aged ≤2years* will be recorded in the eCRF.

4. Clarification on the provision of contraception

Following review of the protocol by the WHO-ERC, it was agreed that the current wording of the pregnancy section would be strengthened.

Changes to the protocol are as follows:

6.2.4. Pregnancy

Female subjects of child-bearing potential, as defined as aged ≥12 years, and sexually active should use barrier contraceptive measures for the duration of the study period. Condoms and spermicide *will* be provided by the investigator / study team, and appropriate counselling should be given to female subjects of child-bearing potential about the risks of becoming pregnant and exposing the foetus to study drugs during the consent process.

5. Clarification of interim analyses planned

Further to a meeting with the chair of the IDMC, it was recommended that the IDMC review the overall risk:benefit of CDA, rather than focus entirely on safety. Therefore the protocol need to be amended to reflect this.

Changes to the protocol are as follows:

11.3.4. Interim analyses

Interim analyses of *efficacy and* safety data are planned as follows:

(1) An Independent Data Monitoring Committee (IDMC) is planned to review the *risk:benefit ratio of CDA, and to achieve this will monitor both efficacy and* safety aspects of this study. The details of the frequency and type of analyses will be described in the IDMC Charter.

6. Reduction in frequency of temperature & parasitology assessments

Feedback from investigators and ethics committees has indicated that 4-hourly temperature measurements and blood film preparation during the in-patient stage of the study is too intensive, especially for younger children. Therefore both assessments will be made at 8-hourly intervals instead of every 4 hours as initially planned.

Changes to the protocol are as follows:

6.2.1. Clinical assessment and vital signs

Tympanic temperature measurements on day 0 at screening and pre-dose & every *8* hours during the in-patient stay until discharge on day 3; single assessments on days 3, 7, 14, 28 and 42.

6.3.1. Parasitology

Microscope blood slides will be prepared on day 0 at screening and pre-dose*, then every 8-hours during the in-patient stay until discharge on day 3. Microscope blood slides will be prepared at each subsequent* visit on days 7, 14, 28 and 42. At each timepoint two thick and one thin film should be prepared. See Study Reference Manual for staining and counting methodology.

Appendix 7: Protocol Amendment 3 changes

Rationale for changes:

1. Withdrawal of the urine lignin test

This protocol amendment is to withdraw the urine lignin test as the test is not sufficiently sensitive nor reproducible in detecting sulfonamide drugs . All patients who have received any unknown antimalarial drug within the past 28 days will be excluded from the study.

5.2.2 Exclusion Criteria

11. Unknown antimalarial drug use within the past 28 days.

6.1 Demographic and Baseline Assessments

Urine test for chloroquine, all subjects (see Study Reference Manual).

6.4 Urine testing for antimalarial drugs

A urine sample will be taken for all subjects to test for the presence of chloroquine.

Appendix 1 – Time and Events Table

Urine test for CQ

2. Change in protocol author and personnel responsible for study coordination

Changes to the protocol are as follows:

Author: Goh,Li Ean, ID MDC; Duparc,Stephan, ID MDC
Study Physician: Dr Allan Pamba
Study Leaders: Ammar Qureshi; Dr. Cletus Ugwuegbulam

3. Changes to Section 6.3.4 -Adequate Clinical and Parasitological Response i.e.:

- **Provision of WHO reference for severe malaria**
- **Addition of an early treatment failure definition**
- **Correction in temperature for ETF, LCF & LPF assessments.**

6.3.4 Adequate Clinical and Parasitological Response

Early Treatment Failure (ETF):

- Development of danger signs or severe malaria on Day 1, 2 or 3 (as defined in World Health Organisation. Severe falciparum malaria. *Trans R Soc Trop Med Hyg*, 2000; **94**, supplement 1) in the presence of parasitaemia
- Parasitaemia on Day 2 higher than Day 0 count, irrespective of tympanic temperature
- Parasitaemia on Day 3 with tympanic temperature ≥ 38.0°C
- Parasitaemia on Day 3 ≥ 25% of count on Day 0

Late Clinical Failure (LCF):

- Development of danger signs or severe malaria after Day 3 in the presence of parasitaemia, without previously meeting any of the criteria of ETF
- Presence of parasitaemia and tympanic temperature ≥ 38.0°C on any day from Day 4 to Day 42, without previously meeting any of the criteria of ETF

Late Parasitological Failure (LPF):

- Presence of parasitaemia on Day 14, 28 or 42 and tympanic temperature < 38.0°C, without previously meeting any of the criteria of ETF or LCF

Appendix 8: Protocol Amendment 04

1. Removal of tertiary objective and endpoint

Rationale:

Tertiary objective and endpoint considered not essential to support primary and secondary endpoints. For logistical reasons this descriptive and exploratory analysis has been removed and will be conducted separately

Changes to the protocol are as follows:

Removal of:

Section 2.3

Tertiary objective

- To investigate known parasitological markers of resistance in subjects with a recrudescence of their initial malaria infection.

Section 3.3

Tertiary efficacy endpoint

- Summarise the incidence of the known markers of resistance in the P.falciparum DHFR and DHPS genes for CDA and Pfmdr gene for lumefantrine, of subjects with recrudescence of their initial infection, by treatment group.

2. Rationale for changes to section 6.3.4 -Adequate Clinical and Parasitological Response i.e.:

- Provision of WHO reference for severe malaria
- Addition of an early treatment failure definition
- Correction in temperature for ETF, LCF & LPF assessments
- Correction of definition of LPF to be in accordance with WHO Guidelines for the Treatment of Malaria (Sept 2005)

6.3.4 Adequate Clinical and Parasitological Response

Changed from:

Subjects will also be assessed for this secondary endpoint against the following definitions and described as either having an Adequate Clinical Parasitological Response (ACPR), early treatment failure, late parasitological failure, late clinical failure, or being not assessable, **on days 14 and 28**.

To:

Subjects will also be assessed for this secondary endpoint against the following definitions and described as either having an Adequate Clinical Parasitological Response (ACPR), early treatment failure, late parasitological failure, late clinical failure, or being not assessable, **on day 28**.

Early Treatment Failure (ETF):

Changed from:

- Parasitaemia on Day 2 higher than Day 0 count, irrespective of tympanic temperature
- Parasitaemia on Day 3 with tympanic temperature ≥ 38.0°C

To:

- Parasitaemia on Day 2 higher than Day 0 count, irrespective of tympanic temperature
- Parasitaemia on Day 3 with tympanic temperature ≥ 38.0°C
- **<u>Parasitaemia on Day 3 ≥ 25% of count on Day 0</u>**

Late Clinical Failure (LCF):

Changed from:

- Presence of parasitaemia and tympanic temperature ≥ 38.0°C on any day from Day 4 to Day 28, without previously meeting any of the criteria of ETF

To:

- Presence of parasitaemia and tympanic temperature <u>≥ 38.0°C</u> (or history of fever) on any day from Day 4 to Day 28 without previously meeting any of the criteria of ETF

Late Parasitological Failure (LPF):

Changed from:

- Presence of parasitaemia on Day 28 and tympanic temperature < 38.0°C, without previously meeting any of the criteria of ETF or LCF

To:

- Presence of parasitaemia on any day from Day 7 to Day 28 and tympanic temperature < 38.0°C, without previously meeting any of the criteria of ETF or LCF

3. Changes to Section 11 – Data Analysis and Statistical Considerations:

Rationale for changes to Section 11:

- Add a detailed discussion of the choice of non-inferiority margin in line with EMEA Guideline (EMEA/CPMP/EWP/2158/99)
- Change the analysis population for the powered key secondary analysis (superiority of CDA vs Coartem in % of patients with parasites at 24 hours) from Per Protocol to ITT in line with EMEA Guideline (EMEA/CPMP/EWP/482/99) and ICH Harmonised Tripartite Guideline (ICH E9)
- Update Missing Data section to reflect ITT and Per Protocol principles
- Amend proposed adverse event reporting to exclude events which are PCR-confirmed recrudescences from most planned outputs
- Minor changes to text for purposes of clarity

Section 11.2 Study design considerations

Addition of new section, 11.2.1, to existing text.

Section 11.2.1 – Sample Size Assumptions

Changed from:

A total of 650 evaluable patients in the CDA group and 325 evaluable patients in the artemether-lumefantrine group will provide **at least** 90% power to show non-inferiority of CDA to artemether-lumefantrine in terms of a one-sided hypothesis test with a 2.5% significance level for the primary endpoint of the parasitological cure rate at Day 28 (PCR corrected). This is based on using a 7% non-inferiority margin and a 2:1 allocation ratio, assuming 93% efficacy for CDA and 95% efficacy for artemether-lumefantrine amongst patients evaluable for the primary per-protocol population. **(Similar efficacy rates are anticipated for the key supportive analysis using the Intent-to-Treat (ITT) population.)**

To:

A total of 650 evaluable patients in the CDA group and 325 evaluable patients in the artemether-lumefantrine group will provide **at least** 90% power to show non-inferiority of CDA to artemether-lumefantrine in terms of a one-sided hypothesis test with a 2.5%

significance level for the primary endpoint of the parasitological cure rate at Day 28 (PCR corrected). This is based on using a 7% non-inferiority margin and a 2:1 allocation ratio, assuming 93% efficacy for CDA and 95% efficacy for artemether-lumefantrine amongst patients evaluable for the primary per-protocol population.

Changed from:

The Day 28 PCR corrected Parasitological Cure Rate of artemether-lumefantrine is estimated to be 95% with values in the literature ranging between 93.5% to 97.5%. These parasitological cure rates have been observed in different trials run in different regions of the world at different times and in different populations. It will be concluded that CDA is non-inferior to artemether-lumefantrine, in terms of parasitological cure rate at Day 28, if the lower bound of the two-sided 95% confidence interval around the treatment difference (CDA minus artemether-lumefantrine) is no less than –7%. This margin of 7% is considered clinically relevant for this patient population and disease area. A standard margin for non-inferiority studies has not been adopted for this therapeutic area at this time. A margin of 10% was considered too wide to be clinically meaningful, while a margin of 5% was considered too restrictive for important therapies in this disease area, a margin of 7% was believed to be clinically meaningful.

To:

At the time of sample size calculation for this study, the Day 28 PCR corrected Parasitological Cure Rate of artemether-lumefantrine was estimated to be 95% with values in the literature ranging between 93.5% to 97.5%. These parasitological cure rates have been observed in different trials run in different regions of the world at different times and in different populations.

11.3.1 Data Analysis Considerations

Changed from:

The following populations will be evaluated:

- Intent to treat (baseline, supportive efficacy, safety analyses)
- Day 28 Per Protocol (efficacy analyses for day 28 endpoints)
- Day 3 Per Protocol (efficacy analyses for day 3 endpoints)
- Day 14 Per Protocol (efficacy analyses for day 14 endpoints)
- Day 42 Per Protocol (efficacy analyses for day 42 endpoints)
- Pharmacokinetic population

The Day 28 Per Protocol Population will be used for the primary analysis of Parasitological Cure Rate at Day 28. The intent-to-treat (ITT) population will be used for key supportive efficacy analyses of the primary endpoint. For other secondary efficacy analyses the Per Protocol Population (Day 3, 14, 28 or 42) will be used as appropriate for the timepoint being analyzed.

Intent-to-treat population (ITT)

All subjects who were randomised and have received any dose of the study medication, irrespective of whether they vomited, will be included in the intent-to-treat population. The intent-to-treat analysis of efficacy will be supportive to per-protocol analyses and will also be used for safety analyses.

Per-protocol populations (PP)

Subjects will be eligible for the per-protocol efficacy analysis at a particular time point, providing the criteria to be included in the intent-to-treat population have been satisfied and the following apply:

- The subject has completed all efficacy visits required to be evaluable (as specified by the protocol in Section 11.3.5.1.) at the time point for analysis.
- No major protocol violation exists with regard to Inclusion/Exclusion criteria
- No prohibited concomitant medications were taken during the study through to this time point
- The patient is compliant with taking all of the study medication (Subject received all scheduled doses, and if vomited within 30 minutes, was re-dosed and did not vomit.)
- Subjects withdrawing from treatment due to a treatment-related adverse event or due to lack of efficacy will not be excluded from the per-protocol population provided that they were compliant prior to withdrawal. Also subjects re-treated with another anti-malarial due to a recrudescence of their baseline infection (confirmed following PCR analysis), will not be excluded. These subjects will be included in the analyses and considered as failures

To:

The following populations will be evaluated:

- Intent to treat
- Per Protocol
- Pharmacokinetic population

Intent-to-treat population (ITT)

All subjects who were randomised and have received any dose of the study medication, irrespective of whether they vomited, will be included in the intent-to-treat population. The intent-to-treat analysis of efficacy will be a supportive analysis population for the primary efficacy endpoint and will also be used for all secondary efficacy and safety analyses.

Per-protocol populations (PP)

The Per Protocol (PP) population will consist of all subjects in the ITT population not identified as major protocol violators. The major protocol violations for which patients would be excluded from the PP population will be described in the Reporting and Analysis Plan and will represent violations that may impact the efficacy endpoints for which non-inferiority comparisons are being made. The decision to exclude a subject from the PP population will be made prior to breaking the blind. This population will be used for primary analysis of the day 28 cure rate and supportive analysis of the secondary efficacy endpoints.

11.3.2 Data Analysis Sets

Changed from:

Data sets will contain a flag to identify for which analysis populations subjects are eligible. The primary time point of interest is day 28 and for the primary analysis of parasitological cure rate, the data set containing subjects evaluable for this analysis will be used (i.e. those having parasite count at day 0, day 3-7, and day 28 and that have not taken another anti-malarial for treatment of a re-infection).

To:

Data sets will contain a flag to identify for which analysis populations subjects are eligible. **Additionally, modified population datasets will be created for the purposes of sensitivity analyses. Full details of these analysis datasets will be given in the Reporting Analysis Plan.**

11.3.3.1 Primary Comparisons of Interest

Changed from:

The comparison will be made for CDA minus artemether-lumefantrine and a two-sided 95% confidence interval for the treatment difference will be used, giving a nominal significance level for falsely rejecting the null hypothesis of 2.5%. As only one comparison is being studied, no adjustment for multiple comparisons is required.

Changed to:

The comparison will be made for CDA minus artemether-lumefantrine and a two-sided 95% confidence interval for the treatment difference will be used, giving a nominal significance level for falsely rejecting the null hypothesis of 2.5%. As only one comparison is being studied, no adjustment for multiple comparisons is required. **Non-inferiority will be concluded if the lower bound of the two-sided 95% confidence interval is no less than than -7%.**

11.3.5.2 Missing data

Changed from:

As described in section 11.3.5.1, subjects will be required to have assessments at all of the key timepoints (day 0, at least one assessment day 3-7, and day 28) to be assessed for the primary analysis. If the patient has parasites present at day 28, then the day 3-7 assessment is not required to determine outcome.

Patients with missing data will be excluded from analysis, with the exception of patients that have been withdrawn due to an adverse event or lack of efficacy; or patients that present with a positive parasite count at any intermittent timepoint day 7 to day 28. These patients will be classified as a failure for the analysis, unless a PCR-corrected value determines that their positive parasite count is a re-infection (new infection). In this case they will be excluded from the analysis rather than considered as a failure.

To:

For the ITT population, subjects will not be excluded from any statistical analyses with the exception of efficacy analyses for PCR-confirmed cases of new malarial infection. For the primary endpoint for this population, an imputation of treatment failure will be made for subjects without a day 28 parasitological assessment. However, all data summaries of the ITT population will be performed with no replacement of missing CRF efficacy and safety data. Patients with missing data at the relevant timepoint will be excluded from analyses for the Per Protocol population eg patients withdrawing from the study prior to day 28 will be excluded from the primary endpoint analysis for the Per Protocol population. Full details of how missing CRF data will be handled will be described in the Reporting Analysis Plan.

Section 11.3.5.7 Efficacy Analyses

Changed from:

A key supportive analysis will be the Intent-to-Treat analysis of the Parasitological Cure Rate (PCR-corrected) at Day 28, with missing data excluded from the analysis

To:

A key supportive analysis will be the Intent-to-Treat analysis of the Parasitological Cure Rate (PCR-corrected) at Day 28, **with missing data imputed as treatment failure**

Changed from:

- Intent-to-Treat analysis of the Parasitological Cure Rate (PCR-corrected) at Day 28, with missing data included as failure.

To:

- Intent-to-Treat analysis of the Parasitological Cure Rate (PCR-corrected) at Day 28, with missing **data excluded**.

Section 11.3.5.8.2 Adverse Events

Changed from:

All treatment-emergent AE's (i.e. with onset time on or after that of first dose), will be reported, with the following summary tables to compare the AE profiles of the two treatment groups for the intent-to-treat population:

To:

All treatment-emergent AE's (i.e. with onset time on or after that of first dose), will be listed and a summary table will be prepared showing the incidence of all recorded AEs by treatment group. However, all other AE tabulations will be produced excluding occurrences of PCR-confirmed P. fulciparum recrudescence. Such events will be classified as treatment failures for the purposes of efficacy analyses but will not be considered AEs for the purposes of comparing the AE profiles between the two treatment groups. Occurrences of new malarial infection, as confirmed by PCR will remain as adverse events and will be reported in all the AE outputs. The following summary tables will be produced for all AEs except those confirmed as recrudescences:

4. Section 13 References

Addition of new references to Section 13 relevant to the provision of the WHO reference for Adequate Clinical and Parasitological Response and choice of non-inferiority margin

The following references have been added:

Anorlu RI, Odum CU, Essien EE. Asymptomatic malaria parasitaemia in pregnant women at booking in a primary health care facility in a periurban community in Lagos, Nigeria. Afr J Med Med Sci. 2001;30 Suppl:39-41

Chandler CI, Drakeley CJ, Reyburn H, Carneiro I. The effect of altitude on parasite density case definitions for malaria in northeastern Tanzania. Trop Med Int Health. 2006 Aug;11(8):1178-84.

Chlorproguanil,dapsone,artesunate (CDA) Investigator's Brochure GM2002/00293/01

Hamour S, Melaku Y, Keus K, Wambugu J, Atkin S, Montgomery J, Ford N, Hook C, Checchi F. Malaria in the Nuba Mountains of Sudan: baseline genotypic resistance and efficacy of the artesunate plus sulfadoxine-pyrimethamine and artesunate plus amodiaquine combinations. Trans R Soc Trop Med Hyg. 2005 Jul;99(7):548-54.

Mueller EA, van Vugt M, Kirch W, Andriano K, Hunt P, de Palacios PI. Efficacy and safety of the six-dose regimen of artemether-lumefantrine for treatment of uncomplicated Plasmodium falciparum malaria in adolescents and adults: a pooled analysis of individual patient data from randomized clinical trials. Acta Trop. 2006 Nov;100(1-2):41-53. Epub 2006 Oct 12.

Njama-Meya D, Kamya MR, Dorsey G. Asymptomatic parasitaemia as a risk factor for symptomatic malaria in a cohort of Ugandan children. Trop Med Int Health. 2004 Aug;9(8):862-8

Okocha EC, Ibeh CC, Ele PU, Ibeh NC. The prevalence of malaria parasitaemia in blood donors in a Nigerian teaching hospital. J Vector Borne Dis. 2005 Mar;42(1):21-4.

Oyakhirome S, Potschke M, Schwarz NG, Dornemann J, Laengin M, Salazar CO, Lell B, Kun JF, Kremsner PG, Grobusch MP. Artesunate--amodiaquine combination therapy for falciparum malaria in young Gabonese children. Malar J. 2007 Mar 12;6:29.

van den Broek I, Kitz C, Al Attas S, Libama F, Balasegaram M, Guthmann JP. Efficacy of three artemisinin combination therapies for the treatment of uncomplicated Plasmodium falciparum malaria in the Republic of Congo. Malar J. 2006 Nov 24;5:113.

5. Appendix 2 – Country Specific Requirements

Protocol text changed from ‘See Appendix 5’ to ‘None’.

Rationale for change:

Appendix 5 refers to the protocol changes made in protocol amendment 1 and hence is incorrectly referenced under Country Specific Requirements. There are no country specific requirements for the territories in which this study is being conducted. Appendix 2 has been updated to reflect the such.
